# Image-based epigenetic profiling with deep learning and high-speed super-resolution microscopy

**DOI:** 10.1186/s13072-026-00662-5

**Published:** 2026-02-28

**Authors:** Yicheng Wang, Nur Syatila Ab Ghani, Munmee Dutta, Shungo Adachi, Kaoru Katoh, Masakazu Namihira, Toutai Mitsuyama, Yutaka Saito

**Affiliations:** 1 Graduate School of Frontier Sciences, The University of Tokyo, 5-1-5 Kashiwanoha, Kashiwa, 277-0882 Chiba Japan; 2 Department of Data Science, School of Frontier Engineering, Kitasato University, 1-15-1 Kitazato, Minami-ku, Sagamihara, 252-0373 Kanagawa Japan; 3 Artificial Intelligence Research Center, National Institute of Advanced Industrial Science and Technology (AIST), 2-4-7 Aomi, Koto-ku, Tokyo, 135-0064 Japan; 4 Department of Proteomics, National Cancer Center Research Institute, Tsukiji 5-1-1, Chuo-ku, Tokyo, 104-0045 Japan; 5 Exploratory Research Center on Life and Living Systems, National Institutes of Natural Sciences, 5-1 Higashiyama Myodaijicho, Okazaki, 444-8787 Aichi Japan; 6 Molecular Biosystems Research Institute, National Institute of Advanced Industrial Science and Technology (AIST), 1-1-1 Higashi, Tsukuba, 305-8566 Ibaraki Japan; 7 Laboratory of Neural Regeneration and Brain Repair, Division of Biological Science, Graduate School of Science and Technology, Nara Institute of Science and Technology (NAIST), 8916-5 Takayama-cho, Ikoma, Nara, 630-0192 Japan

**Keywords:** Image-based epigenetic profiling, Super-resolution microscopy, Deep learning, Histone deacetylase inhibitor, Rett syndrome

## Abstract

**Background:**

Comprehensive profiling of epigenetic states is essential for understanding gene regulation and disease mechanisms. Sequencing-based methods such as ChIP-seq, Hi-C, and RNA-seq provide genome-wide views of histone modifications and 3D genome organization, but lack spatial resolution within single nuclei.

**Results:**

Here we present an image-based epigenetic profiling framework that combines high-speed super-resolution microscopy with deep learning. Using models of (i) histone deacetylase inhibition in HEK293T cells and (ii) Rett syndrome iPS cells carrying *MECP2* mutations, our approach accurately discriminated their epigenetic states (99.6% and 96.1% accuracy, respectively) and identified the nuclear periphery as a hotspot of H3K27ac and CTCF redistribution. Sequencing-based analyses showed compartment switching and lamina-associated domain alterations consistent with the image-based features. These results demonstrate that high-speed super-resolution imaging, when combined with deep learning, provides a powerful tool for epigenetic profiling.

**Conclusions:**

Our framework offers a generalizable strategy for image-based epigenetic profiling to uncover chromatin alterations in development, disease, and therapeutic response.

**Supplementary Information:**

The online version contains supplementary material available at 10.1186/s13072-026-00662-5.

## Background

 Genomic DNA within a cell nucleus is hierarchically packaged into higher-order chromatin structure through protein-DNA interactions [1]. Previous studies revealed the importance of chromatin organization for regulating gene expression and the proper functioning of living cells [2, 3]. Many cellular activities are significantly impacted by the disruption of chromatin organization, which is frequently linked to biological processes like aging and disease [4–7]. Epigenetic modifications such as DNA methylation and histone modifications introduce changes to the compaction of chromatin structure in response to mutations or environmental fluctuations, which alter the gene expression and subsequent biological processes [8, 9]. The histone modification mark H3K27ac, which represents the acetylation of H3 at lysine 27, is commonly associated with active enhancers and promoters, serving as a key indicator of regions with active transcription [10]. In parallel, the CCCTC-binding factor (CTCF) functions as a key architectural protein that regulates chromatin organization and long-range genomic interactions, by acting either as a transcriptional repressor or activator [1, 3]. Together, these epigenetic signatures coordinate gene expression and define the regulatory landscape of the genome.

Profiling of epigenetic information in various cell types and individuals has provided insights into the mechanisms of epigenetic regulation [11]. Sequencing-based epigenetic profiling techniques have been developed to analyze the detailed higher-order structure of chromatin. One of these, high-throughput chromatin conformation capture (Hi-C) has revealed the segregation of the genome into active and inactive compartments (referred to as A and B compartments, respectively) [12–14]. At higher resolution, Hi-C can detect highly self-interacting chromatin domains called topologically associated domains (TADs) [12, 14, 15] and long-range interactions between regulatory elements in the form of chromatin loops [14, 16]. Chromatin immunoprecipitation with high-throughput sequencing (ChIP-seq) allows us to study histone modifications and transcription factor binding [17, 18]. RNA-seq profiles the gene expression changes between different conditions or individuals, allowing us to identify genes altered by epigenetic changes. However, profiling of the chromatin changes and epigenetic modifications through these sequencing-based techniques has only been limited to fixed cells, thus disregarding the dynamic feature of higher-order chromatin structure (19, 20). While sequencing-based methods provide genome-wide insights into chromatin accessibility and binding profiles, they are limited in their ability to capture changes in physical size, organization or spatial localization of chromatin sub-structures that occur in response to biological perturbations such as genetic mutations or diseases. To address these limitations, image-based methods using microscopy have been used as another significant approach for epigenetic profiling.

Imaging technologies allow the visualization of chromatin in living cells while the observation of detailed chromatin structures is difficult due to the resolution limit of optical microscopy (around 200–300 nm) [19, 20]. Recent advances in super-resolution microscopy (SRM) have improved the resolution limit of optical microscopy to be around 100 nm or even less. Several studies used SRM to observe the spatial relationship between epigenetic modifications and DNA compaction [4, 8, 21–23]. Xu and colleagues [22] used stochastic optical reconstruction microscopy (STORM) to categorize chromatin states into three distinct types of nucleosome aggregates. Cremer et al. [23] described 3D nuclear topography of active and inactive nuclear compartments based on chromatin compaction levels using 3D fluorescence in situ hybridization (3D-FISH) and 3D structured illumination microscopy (3D-SIM).

An increasing number of studies show that deep learning exhibits better robustness and stronger ability in cell image analysis compared to traditional image processing techniques [24]. Deep learning has been applied to various image-based tasks including the segmentation of cell regions, the classification of cell types [24], and the detection of image-based biomarkers in tumor progression [4] and cellular senescence [25]. However, these applications have not been extensively studied for SRM due to the low throughput of typical SRM that makes it difficult to obtain large image datasets necessary for deep learning.

Here, we employ a high-speed SRM (Yokogawa CSU-W1 SoRa) [26], which can capture around 120 nm super-resolution images up to 20 frames per second. The Yokogawa CSU-W1 SoRa system extends conventional spinning-disk confocal microscopy by incorporating an optical design that sharpens the effective point spread function prior to computational processing. Through a modified disk architecture and subsequent image reconstruction, emitted fluorescence signals are reassigned and refined, enabling lateral resolution beyond that of standard confocal imaging [26]. This system has been successfully applied in a variety of biological imaging studies [27–32], demonstrating its advantages in resolving subcellular structures and dynamic processes, including actin-dependent membrane polarization during asymmetric division in *Drosophila* neuroblasts [27], cytoskeletal remodeling in macrophages [28], protein sorting within the Golgi apparatus [29], endosomal membrane tubulation [30], multiplexed live-cell organelle imaging with deep learning–based segmentation [31], and mitochondrial morphological remodeling associated with neuronal activity and sleep regulation [32].

By combining this high-speed SRM and deep learning, we present an image-based epigenetic profiling system for cell classification and image feature discovery. We assess the effectiveness of this approach in investigating epigenetic changes induced by two distinct biological perturbations.

For the first case study, we treated human embryonic kidney 293 (HEK293T) cells with or without valproic acid (VPA). Valproic acid (VPA) is an HDAC inhibitor that targets the active site of Class I and IIa HDACs [33, 34], which prevents the removal of acetyl groups from histones. HDAC inhibition is known to cause increased acetylation of histones, which modulates chromatin decompaction and transcriptional activation [10, 35]. The drug is primarily used to treat neurological conditions like epilepsy, migraine and bipolar disorders [33] and has recently gained attention for its therapeutic applications in cancer [36, 37]. HDAC inhibition directly alters the acetylation landscape and affects chromatin compaction, providing an ideal case study for evaluating our framework.

For the second case study, we analyzed Rett syndrome (RTT) iPS cells harboring *MECP2* gene mutations, specifically in the methyl-CpG-binding domain (MBD) and transcriptional repression domain (TRD). Rett syndrome is a rare neurodevelopmental disorder that predominantly affects young females, caused by X-linked Methyl-CpG binding protein 2 (*MECP2*) gene mutation that disrupts normal brain development and function. The MECP2 protein plays a crucial role in regulating gene expression and chromatin structure (38–41). The MECP2’s MDB is responsible for the methylated DNA binding while the TRD domain is associated with transcriptional repression of *MECP2* target genes [38]. Mutations to these two important domains may affect chromatin organization.

Our framework successfully discriminated the epigenetic states of VPA-treated cells and RTT cells from their control, with accuracies of 99.6% and 96.1%, respectively. The analysis of image features that contributed to the discrimination suggested the nuclear periphery as a hotspot of epigenetic changes, and the colocalization of H3K27ac and CTCF foci characteristic in each biological system. Sequencing-based epigenomic profiling using ChIP-seq, HiC and RNA-seq showed compartment switching and lamina-associated domain alterations by VPA treatment, being consistent with the image-based features.

## Methods

VPA-treated HEK293T and RTT iPS cells are collectively referred to as perturbed cells, while untreated HEK293T and healthy iPS cells are referred to as control cells throughout this study.

### Cell culture and treatments

All cell cultures used in this study were provided by the RIKEN BRC through the National BioResource Project of the MEXT, Japan. VPA treatment was performed on HEK293T cells. The cells were cultured in DMEM (Nacalai Tesque) containing 10% fetal bovine serum (FBS) (Biowest) and 10 µg/ml gentamicine (Nacalai Tesque). For each experiment, HEK293T cells were replaced in a fresh DMEM medium containing 10% FBS with or without 1 mM VPA and cultured for one day. Human-induced pluripotent stem cells from a healthy individual (HPS0076) and from Rett syndrome patients (HPS3042, HPS3049 and HPS3084) were cultured under feeder-free conditions using mTeSR™ Plus medium (VERITAS, #ST-100–0276) on tissue culture-treated plates coated with hESC-qualified Matrigel^®^ (Corning, #354277), according to the manufacturer’s instructions [39, 40]. Cells were maintained at 37 °C in a humidified incubator with 5% CO₂ and passaged every 5–7 days using Versene Solution (Gibco, # 15040066). The medium was changed daily. For ChIP-seq, those iPS cells cultured in 10 cm dishes coated with Matrigel were used.

Both control and perturbed cells were washed with PBS, fixed in 4% paraformaldehyde in PBS, and stained with the indirect fluorescent antibody method: primary antibodies were anti-H3K27ac IgG (308–34843, MAB Institute, Inc.) and anti-CTCF IgG (3418 S, Cell Signaling Technology); the secondary antibodies were CF488 donkey anti-rabbit IgG (H + L), highly cross-adsorbed (Biotium, 20015) and CF568 donkey anti-mouse IgG (H + L), highly cross-adsorbed (Biotium, 20105). Hoechst33342 (Dojindo laboratories) was used for nuclear DNA staining.

### High-speed super-resolution image aquisition

Cells were imaged by Yokogawa CSU-W1 SoRa super-resolution spinning disc confocal system (Tokyo, Japan) [26]. SoRa spinning disk was equipped with microlens attached to the 50 μm pinhole disk to pick up the super-resolution component of diffracted light. The wave lengths of laser lines for excitation sources were 405, 488, 561 and 640 nm. The resolution was 150 nm when the image was acquired with a CCD camera. After applying deconvolution, the resolution of about 120 nm was achieved. The size of one pixel corresponded to around 20 nm. Individual images were represented in three channels: H3K27ac (red), CTCF (green), and nuclear DNA (blue) with size of 2306 × 2306 pixels. An example image is shown in Figure S1.

### Image preprocessing and single-cell segmentation

Mask R-CNN [41] was employed to segment individual cell nuclei from multi-cell images. This model has demonstrated strong performance in handling challenging segmentation tasks, such as separating overlapping nuclei and identifying nuclei with ambiguous boundaries [42]. We trained the Mask R-CNN using the 2018 Data Science Bowl dataset [43], which includes images of diverse cell types, magnifications, and imaging modalities.

To adapt the trained model to our data, super-resolution SoRa images (2306 × 2306 pixels) were down-sampled to 512 × 512 using OpenCV [44] to match the dimensions of the training data. The DNA channel of the down-sampled image was used as input for segmentation. Predicted masks were then resized back to the original resolution and applied to the full-size images to extract individual nuclei.

Post-processing was performed to filter out low-quality or outlier segments based on the following criteria: box aspect ratio *z*-score > 1.5, each side length out of 200 to 600 pixels, pixel area > 30,000, or roundness < 0.7. This pipeline yielded 3,046 and 3,516 segmented nuclei for the untreated and VPA-treated conditions, and 3,595 and 1,723 nuclei for control and RTT cells. Representative segmentation results are shown in Figure S2. Manual inspection confirmed that the majority of cells were correctly segmented. Finally, zero padding was performed around each image so that all images had the same size of 600 × 600, which were used as input to the CNN-based classification model.

### Deep learning–based classification of epigenetic States

Four types of images were used for training the classification models: H3K27ac-only, CTCF-only, DNA-only, and three-channel composite images containing all markers. For the single-channel cases, the same image was duplicated across all three RGB channels to generate pseudo–three-channel inputs. The dataset was split into training and validation sets at a ratio of 80:20.

Three convolutional neural network (CNN) architectures were evaluated. The first was a 9-layer ResNet with one fully connected layer (referred to as the 9Conv + 1 F model; Fig. 2a). The second architecture added a global average pooling layer before the final classification layer (Figure S3a). The third was a deeper ResNet model with 17 convolutional layers, a global average pooling layer, and a fully connected layer (Figure S3b). All models used residual blocks and shortcut connections. Model architectures were implemented using the PyTorch library [45], with base components from the torch.nn module.

To accommodate the physical resolution of the input images, the kernel size of the first convolutional layer was chosen based on spatial sampling constraints. Each image pixel corresponded to approximately 50 nm in real space, while the effective optical resolution of the imaging system was about 120 nm. Therefore, any resolvable structure would span at least 2–3 pixels, suggesting that the kernel size should be larger than 2 × 2 or 3 × 3. In this study, we adopted a 7 × 7 kernel so that the model captured not only the core signal but also surrounding contexts, which could be important for recognizing subtle spatial patterns in chromatin architectures. All models were initially pre-trained on the COCO dataset, which contains over 330,000 images spanning 80 object categories [46]. Fine-tuning was then performed using our own image data to classify epigenetic states (control vs. perturbed cells). The fine-tuning process used the Adam optimizer with binary cross-entropy loss, an initial learning rate of 1e − 4 (decayed to 1e − 6), a batch size of 128, and 300 training epochs.

### Score-CAM–based visualization of discriminative nuclear regions

To interpret our CNN-based models and identify image-based features contributed to discriminating epigenetic states, we employed Score-CAM [47]. Score-CAM generates a visual heatmap that highlights regions within an input image contributing most to the model’s prediction. It operates by linearly combining activation maps obtained from scaled forward passes through the network. We implemented Score-CAM using the torchcam library [48], and generated corresponding heatmaps for each image, which were used for downstream analysis.

To compute average Score-CAM heatmaps for group-level visualization, we applied alignment and normalization procedures to individual heatmaps. Specifically, each nucleus was fitted with an ellipse, and the corresponding Score-CAM heatmap was rotated and scaled so that the major and minor axes of the nucleus were standardized across cells (Figure S4a). This preprocessing step was performed only for generating the average heatmap, to account for variations in cell orientation and size. Individual heatmaps used for downstream quantitative analysis were left in their original coordinate space.

### Quantitative feature extraction from score-CAM heatmaps

To quantitatively analyze Score-CAM heatmaps, we defined two custom metrics that capture nuclear activation patterns:

*Nuclear intensity*: The mean Score-CAM value across all non-zero pixels within the nuclear mask, reflecting the overall activation strength within the nucleus.

$${\mathrm{Nuclear~Intensity}}=\frac{1}{{\left| M \right|}}\mathop \sum \limits_{{p \in M}} S\left( p \right)$$

where *S*(*p*) is the Score-CAM intensity at pixel *p*, and *M* is the set of pixels within the nuclear mask for which *S*(*p*) > 0. 

*Nuclear periphery enrichment*: The nuclear region was divided into five concentric elliptical zones (part1 to part5), from innermost to outermost, based on the fitted ellipse. For each zone, we computed the mean Score-CAM intensity. Part5 corresponds to the nuclear periphery. This metric enabled us to examine spatial distribution patterns of activation signals, with a particular focus on perinuclear changes (Figure S4b).

$${\mathrm{Nuclear~Periphery~Enrichment}}=\frac{{\frac{1}{{\left| {{R_5}} \right|}}\mathop \sum \nolimits_{{p \in {R_5}}} S\left( p \right)}}{{\frac{1}{{\left| M \right|}}\mathop \sum \nolimits_{{p \in M}} S\left( p \right)}}$$

where $\:S\left(p\right)$ is the Score-CAM intensity at pixel $\:p$, $\:{R}_{5}$ is the set of pixels in the outermost region (part5), and $\:M$ is the set of all pixels within the nuclear mask.

In addition to these custom metrics, we extracted a set of over 90 standard image features from the Score-CAM heatmaps using the image analysis package, Histomicstk [49] which contains functions to compute a variety of image-based features that quantify the appearance and/or morphology of an objects/regions in the image. These features span five categories: size (e.g., area, axis length), shape (e.g., roundness, eccentricity), intensity (e.g., mean, standard deviation), edge features (e.g., Canny edge count), and texture descriptors (e.g., Haralick features, entropy). For each feature, we performed *t*-tests between perturbed and control groups using the ttest_ind function from the scipy library [50]. Features with *p* < 1e − 2 were visualized in histograms grouped by feature category, providing which feature categories showed significant changes in perturbed conditions.

### Nuclear puncta segmentation

To segment nuclear puncta, defined as bright, discrete foci in the H3K27ac and CTCF super-resolution images, we implemented a custom pipeline combining multi-level intensity thresholding and marker-based watershed segmentation. This approach was designed to robustly isolate chromatin-associated structures across varying nuclear morphologies.

Each single-channel image was first binarized using multi-level Otsu thresholding with four intensity classes, enabling separation of the brightest puncta from background and mid-intensity regions. The Euclidean distance transform was then applied to the binary mask to estimate object separation, and local maxima were identified as candidate puncta centers. These maxima served as markers for a watershed algorithm applied to the inverted distance map, producing a labeled mask of individual puncta. To exclude oversized structures that are unlikely to represent discrete foci, we removed segmented objects with an area greater than 300 pixels.

All steps were implemented in Python using components from the scikit-image, scipy, and numpy libraries. The complete segmentation workflow was encapsulated in a custom function (thre_h_watershed), enabling efficient processing across all samples (Figure S4c). This method allowed consistent extraction of nuclear puncta suitable for downstream morphological and spatial analyses.

### Quantitative feature extraction from nuclear puncta

To quantitatively characterize the segmented nuclear puncta, we computed five metrics representing their abundance, morphology, signal intensity, spatial localization, and inter-marker proximity. 

*Puncta number*: The total number of puncta within a nucleus was defined as the number of connected components identified in the segmentation mask.

*Average puncta area*: The mean area of all segmented puncta (in pixels) was calculated as.

$${\mathrm{Puncta~Area}}=\frac{1}{N}\mathop \sum \limits_{{i=1}}^{N} {A_i}$$

where $\:{A}_{i}$ is the area of the $\:i$-th punctum, and $\:N$ is the total number of puncta.

*Average puncta intensity*: Each punctum’s average fluorescence intensity was computed from the original image, and the mean intensity across all puncta was used.

$${\mathrm{Puncta~Intensity}}=\frac{1}{N}\mathop \sum \limits_{{i=1}}^{N} {I_i}$$

where $\:{I}_{i}$ is the mean intensity of the $\:i$-th punctum.

*Puncta irregularity*: To assess shape irregularity, we computed the elongation ratio of each punctum as the ratio of its major to minor axis lengths, and averaged across the nucleus.

$${\mathrm{Puncta~irregularity}}=\frac{1}{N}\mathop \sum \limits_{{i=1}}^{N} \frac{{L_{i}^{{major}}}}{{L_{i}^{{\hbox{min} or}}}}$$

where $\:{L}_{i}^{\mathrm{major}}$and $\:{L}_{i}^{\mathrm{minor}}$ are the lengths of the major and minor axes of the $\:i$-th punctum.

*Nuclear Spatial distribution*: Each nucleus was divided into five concentric elliptical layers (part1 to part5, from nuclear center to periphery) as Figure S4b. For each layer *j*, the puncta density was calculated as.

$${\mathrm{Puncta~densit}}{{\mathrm{y}}_{{\mathrm{region~j}}}}=\frac{{{n_j} \cdot 250}}{{{A_j}}}$$

where $\:{n}_{j}$ is the number of puncta located in region *j*, and $\:{A}_{j}$ is the area of that region (in pixels). The result represents the number of puncta per 250-pixel unit area, enables assessment of how puncta are distributed across nuclear space.

*Minimum distance between markers (Colocalization)*: To quantify spatial proximity between H3K27ac and CTCF puncta, we measured the minimum Euclidean distance from each H3K27ac punctum centroid $\:{C}_{i}^{\mathrm{(H3K27ac)}}$ to its nearest CTCF punctum centroid$\:{C}_{j}^{\mathrm{(CTCF)}}$.

$$H3K27ac\_To\_CTCF\_MinDis\tan ce=\frac{1}{N}\mathop \sum \limits_{{i=1}}^{N} \mathop {\hbox{min} }\limits_{j} |C_{i}^{{(H3K27ac)}} - C_{j}^{{(CTCF)}}|$$

$$CTCF\_To\_H3K27ac\_MinDis\tan ce=\frac{1}{N}\mathop \sum \limits_{{i=1}}^{N} \mathop {\hbox{min} }\limits_{j} |C_{i}^{{(CTCF)}} - C_{j}^{{(H3K27ac)}}|$$

where centroids are defined as the geometric centers of individual puncta identified by segmentation (Figure S4c). This metric captures local chromatin spatial relationships, potentially reflecting functional interactions or structural colocalization between active regulatory elements (H3K27ac) and architectural anchors (CTCF). *Puncta overlap ratio*: To quantify the spatial overlap between chromatin puncta marked by different epigenetic factors (H3K27ac and CTCF), we computed an overlap ratio based on the geometric proximity of segmented puncta. For each punctum, its centroid and major/minor axis lengths were extracted using region props. Because the segmented puncta typically exhibit an approximately elliptical shape, an effective radius was defined as the average of the half-lengths of the major and minor axes.

$${r_i}~ \approx ~\frac{{L_{i}^{{major}}+~L_{i}^{{\hbox{min} or}}}}{4}$$

Pairwise Euclidean distances between all puncta centroids from the two markers were then calculated. For any punctum pair, geometric overlap was defined when the centroid distance was smaller than or equal to the sum of their effective radii:

$${d_{ij}} \leqslant ~{r_i}+~{r_j}$$

For each punctum from the source marker (e.g., H3K27ac), we evaluated whether it overlapped with at least one punctum from the target marker (e.g., CTCF). The overlap ratio was then defined as the proportion of source puncta that exhibited at least one such overlap.

### Simulating low-resolution fluorescence microscopy by Gaussian blurring

To evaluate the impact of spatial resolution on epigenetic image analysis, we simulated low-resolution fluorescence microscopy images by applying Gaussian blur to the original super-resolution data. Specifically, we used the cv2.GaussianBlur function from the OpenCV library [44] with a kernel size of 11 × 11 and the automatic estimation of standard deviation (sigmaX = 0). The kernel size of 11 was selected to approximate the resolution degradation expected when moving from super-resolution (120 nm effective resolution) to conventional fluorescence microscopy. Given that each image pixel corresponds to approximately 50 nm in physical space, a kernel of size 11 covers a region approximately 550 nm wide, more than four times the original optical resolution. This ensures that fine submicron-scale features such as nuclear puncta associated with chromatin structure are effectively blurred, mimicking the information loss that occurs under conventional resolution. The blurred images were used for both classification model training and downstream morphological analyses, enabling direct comparison to super-resolution results.

### Epigenomic profiling by sequencing

Hi-C sequencing, RNA-seq, H3K27ac and CTCF ChIP-seq experiments were performed on both untreated and VPA-treated HEK293T cells following the manufacturer’s protocol. For healthy and RTT iPS cells, only ChIP-seq was performed.

Hi-C was performed according to the protocol of EpiTect Hi-C Kit from Qiagen, and the libraries were prepared for HEK293T cells treated 1 mM VPA for 1 day and control untreated cells as described in the protocol of EpiTect Hi-C Kit. The sequencing by using Illumina NovaSeq 6000 (Illumina) was performed to obtain paired end reads. For RNA-seq, total RNA was extracted from HEK293T cells treated with 1 mM VPA for 1 day or control untreated cells using TRIzol reagent (Invitrogen) according to the manufacturer’s instructions. The libraries were constructed according to DNBSEQ Eukaryotic Strand-specific mRNA library protocol. The sequencing by using DNBSEQ (MGI) was performed to obtain paired end reads. RNA-seq experiments were performed in triplicate.

ChIP-seq was performed according to the protocol published by Agilent technologies (Santa Clara, CA), with the following modifications. For VPA treatment, the HEK293T cells were treated with 1 mM VPA for 1 day. All control and perturbed cells were fixed by 1% formaldehyde solution for 10 min. After harvesting the fixed cells, the cells were lysed in lysis buffer, and sonicated using Misonix XL2020 sonicator (MISONIX Inc., NY) until the DNA fragments were 200–600 base pairs in length. 3.0% of the total volume was stored as input at − 20 °C, until use. Immunoprecipitation was performed at 4 °C, overnight with antibodies described below. DNA/beads were washed with a low salt buffer (20 mM Tris-HCl pH 7.4, 150 mM NaCl, 2 mM EDTA, 0.1% sodium dodecyl sulfate (SDS), 1% Triton X-100) once, and then further washed with a high-salt buffer (20 mM Tris-HCl pH 7.4, 400 mM NaCl, 2 mM EDTA, 0.1% SDS, 1% Triton X-100) once before washing with a RIPA buffer. Immune complexes were disrupted with direct elution buffer (50 mM Tris-HCl pH8.0, 10 mM EDTA, 1% SDS) and the covalent links between immunoprecipitates and input chromatin were disrupted by incubation at 65 °C for overnight. DNA was further incubated with RNaseA and proteinase K (Nacalai Tesque), purified by phenol extraction, and ethanol precipitated. DNA pellets were dissolved in Tris-EDTA (TE) buffer (10 mM Tris-HCl, 1 mM EDTA pH8.0). The antibodies were used for immunostaining. The sequencing libraries were prepared using the NEBNext ChIP-seq Library Prep Master Mix Set for Illumina (NEB) according to the manufacturer’s instructions. The sequencing by using Illumina NovaSeq 6000 (Illumina) was performed to obtain paired end reads. For the VPA case, ChIP-seq experiments were performed in triplicate. For the RTT case, each RTT patient line (HPS3042, HPS3049, and HPS3084) and the healthy individual line were prepared with two biological replicates, each with up to three technical replicates.

### Sequencing data processing and integrative analyses

Raw Hi-C reads were processed using the Juicer pipeline (v1.9.9) [51] for filtering, mapping and creating contact matrices. Reads were aligned on the hg38 human reference genome. Singleton, low-quality, unmapped, dangling, self-circle paired-end reads, and PCR duplicates were removed after mapping. The matrices were normalized using the square root of vanilla coverage (VC_SQRT) method. Compartment calling at 250-kb resolution was performed using HOMER’s runHiCpca.pl (v4.11) [52]. Differential compartments (compartment switching) were assigned using HOMER’s findHiCCompartments.pl. Differential compartments were defined as regions exhibiting differences in the first eigenvector (PC1) exceeding 0.5 (-diff 0.5). To assess the robustness of this difference threshold (-diff), we evaluated multiple cutoffs (0.3, 0.4, 0.5, 0.6 and 0.7) and compared the resulting differential A/B compartment assignments. The consistency of the compartmentalization patterns across these thresholds supported the reliability of the analysis and provided technical justification for selecting 0.5 as the threshold for identifying differential compartments. TAD calling at 25-kb resolution was performed using HiCExplorer (v3.7.2) [53], and differential TAD detection by TADCompare (v1.10.0) [54].

RNA-seq raw reads were processed with SOAPnuke [55] to remove adapters and low-quality reads. The generated clean read FASTQ files were aligned to the hg38 genome using STAR (v.2.7.10) [56] and the read count values of each gene were obtained using htseq-count (v2.0.3) [57]. Differential gene expression analysis was performed by DESeq2 (v1.40.1) (FDR < 0.05, log2 fold change ≥ 1 or ≤ − 1) [58]. Gene Ontology enrichment analysis was performed by ClusterProfiler (v4.8.1) (*p* < 0.01, *q* < 0.05) [59].

ChIP-seq reads were aligned on the hg38 genome by Bowtie2 (v2.1.0) [60]. The Pearson’s correlation coefficient between conditions and replicates was evaluated using plotCorrelation by deepTools (v3.5.1) [61]. Technical replicates for each biological replicate of the RTT patient lines were first merged using SAMtools [62]. Subsequently, biological replicates from the three RTT lines were combined to generate two biological replicates representing a single RTT group. The same procedure was applied to the healthy individual lines. Peak calling was performed by MACS (v3.0.0) (-g hs -f BAMPE -q 0.05) [63]. Differential peak analysis was performed by the DiffBind R package [64] to obtain consensus peaks across replicates as well as differential peaks, i.e. gained and lost peaks (v3.10.0) (FDR < 0.05, log2 fold change ≥ 1 or ≤ − 1). Bigwig files describing the normalized read coverage were generated using deepTools’ bamCompare by comparing chipped and input samples using default parameters.

ChIP-seq signal distribution across the genome was counted using deepTools’s multiBigwigSummary. Average signal values were calculated across two types of regions: [1] genome-wide average signals using equally sized 10 kb genomic bins; and [2] chromatin regions defined using ChromHMM annotations from ENCODE (HEK293T: ENCFF071AXS; iPS: ENCFF247KQA) [65]. Specifically, we analysed the enrichment of regions annotated as heterochromatin (Het) by ChromHMM, as well as active chromatin regions approximated using ChromHMM-defined transcriptionally active states, including active enhancers (*Enh*) and active promoters (*Tss*) [66]. ChIP-seq read coverage over these regions was used to quantify signal enrichment. For box plot visualization, outliers were removed with the threshold of the first quartile (Q1) minus 1.5 times the interquartile range (IQR), or the third quartile (Q3) plus 1.5 times the IQR (Q1–1.5 × IQR, Q3 + 1.5 × IQR). The outlier removal is solely for visualization; statistical testing was performed with unfiltered data. Motif analysis was performed using HOMER [52].

For VPA-treated and untreated cells, genomic features from Hi-C, RNA-seq and ChIP-seq data were compared with reported DamID data on HEK293 cells from Altemose et al. [67]. The significance of overlap between two genomic regions was calculated using a permutation test by the RegioneR package (v1.3.0) [68].

## Results

### Overview of the study

**Fig. 1:**
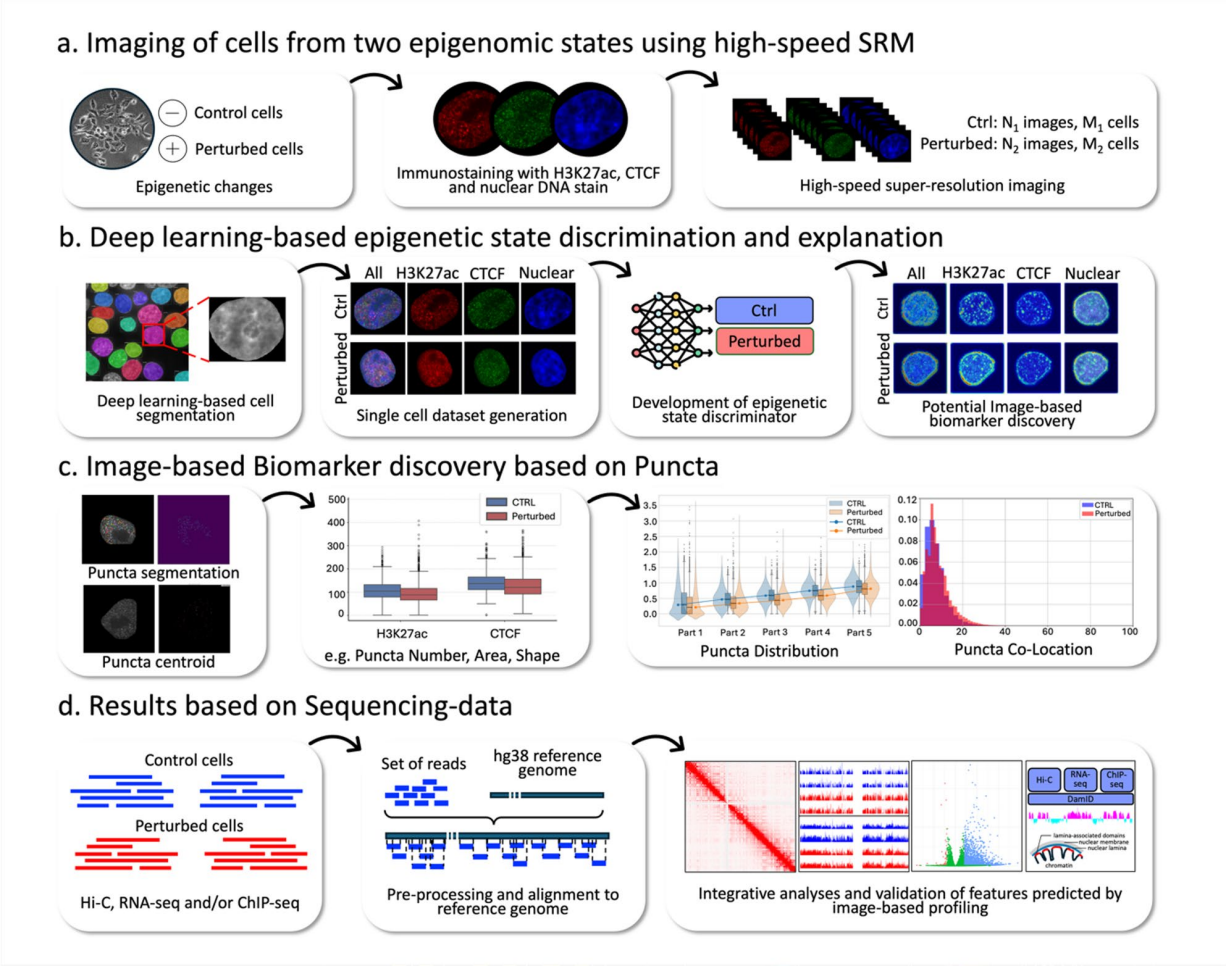
Overview of the study. **a** Image acquisition. Cells from two biological conditions were stained with H3K27ac, CTCF, and nuclear DNA. Super-resolution images were then captured using SoRa microscopy. **b**, **c** Image-based analysis. Acquired images were analyzed using deep learning models for cell segmentation, epigenetic state classification, and quantitative analysis to identify image-based features. **d** Sequencing-based analysis. ChIP-seq, Hi-C, and RNA-seq were performed on cells under the same conditions as those imaged. The sequencing results were compared with the image-based results

The overview of our framework is shown in Fig. 1. The cells from two different conditions were used, designated as Control and Perturbed. The cells in each condition were stained for nuclear DNA (Hoechst), CTCF, and H3K27ac. More than 400 super-resolution images of the cells (above 200 for each condition) were obtained by the high-speed SRM with each staining signal in a different channel (Fig. 1a, Figure S1). Notably, this large number of images was obtained within an hour. These image data were analyzed through the following steps (Fig. 1b-c, Methods).

We segmented individual cell regions from the multi-cell images using the nuclear staining channel, yielding above 6,000 cells in total (Figure S2). Based on these single-cell datasets, we developed a deep learning model to discriminate the epigenetic states of cells (Control vs. Perturbed) (Fig. 1b). We then identified image-based features of the epigenetic states using the trained classification model. In addition, we further extracted nuclear puncta (defined as bright, discrete foci in the H3K27ac and CTCF super-resolution images) and performed quantitative analysis to find image-based features characterizing the epigenetic states (Fig. 1c).

Sequencing-based epigenomic analyses (Fig. 1d) were conducted to compare imaging and sequencing data, providing a more holistic view of the epigenetic landscape.

We performed two case studies: [1] VPA-treated HEK293T cells as the perturbed condition and untreated HEK293T cells as the control, and [2] iPS cells from RTT patients as the perturbed condition and iPS cells from healthy individuals as the control.

### Image-Based analysis of VPA-Treated cells reveals epigenetic signal changes

**Fig. 2:**
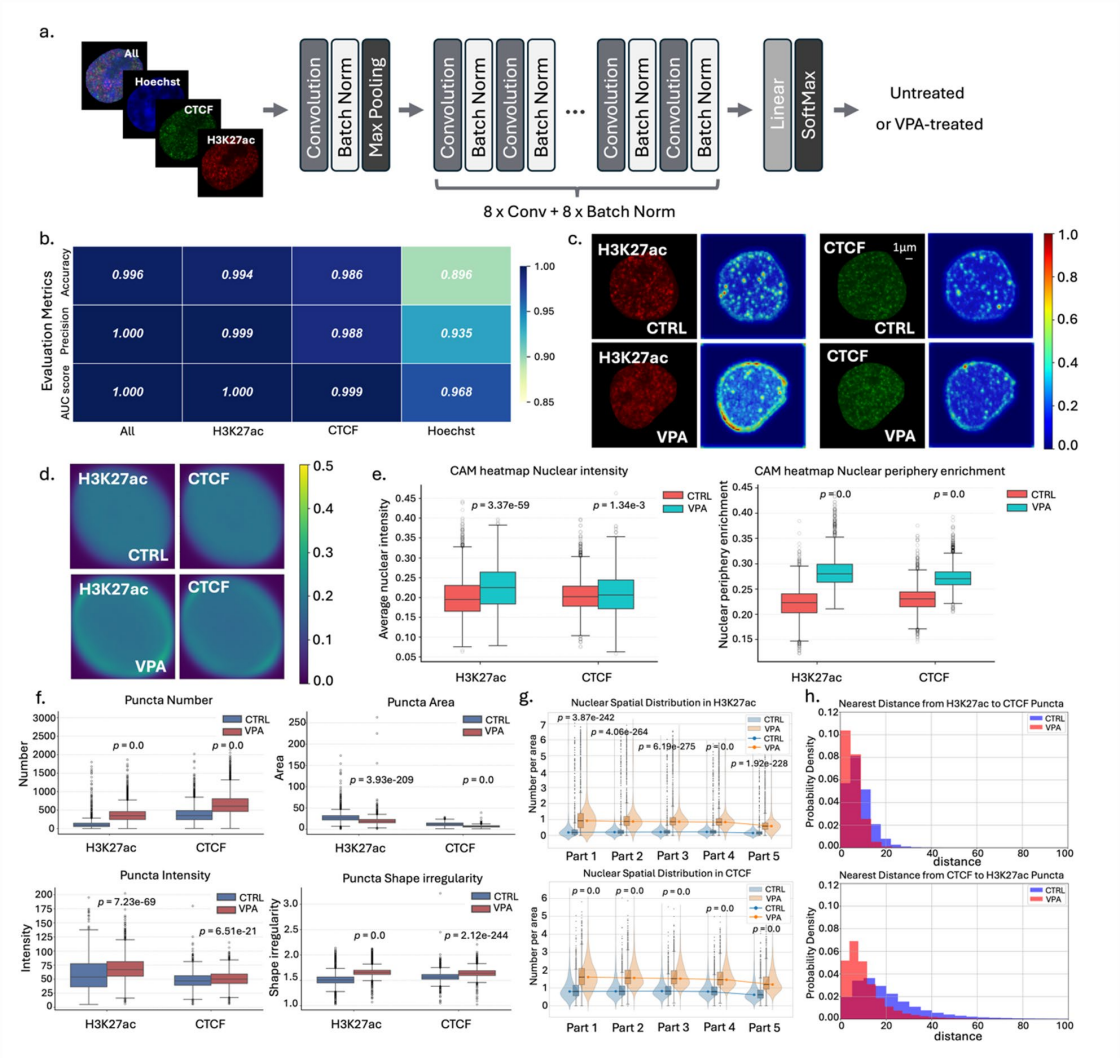
Image-based epigenetic analysis in VPA-treated cells. **a** Architecture of the CNN model for cell classification. The 9Conv + 1 F model consists of nine convolutional (Conv) layers, nine batch normalization (Batch Norm) layers, and one fully connected layer. This model was used for subsequent analyses. The two alternative models are shown in Figure S3a, b. **b** Performance of the 9Conv + 1 F classification model. Model performance was evaluated using accuracy, precision, and AUC. The performance of the two alternative models is presented in Figure S3c. **c** Representative Score-CAM heatmaps. Examples of an untreated (CTRL) and a VPA-treated cell with their corresponding Score-CAM heatmaps under H3K27ac and CTCF staining. Red regions indicate areas contributing most strongly to classification. **d** Averaged Score-CAM heatmap after normalization. To standardize different cell angles and sizes, Score-CAM heatmaps were aligned based on their fitted ellipses (Methods). **e** Quantitative analysis of Score-CAM heatmap nuclear intensity and nuclear periphery enrichment. **f**, **h** Quantitative analysis of nuclear puncta (examples were shown in Figure S4c), including number, area, intensity, shape, distribution, and colocalization. Statistical testing was performed using a paired two-tailed Student’s *t*-test

### Machine learning models to discriminate VPA-treated and untreated cells

We developed convolutional neural network (CNN) models to discriminate epigenetic states between VPA-treated and untreated cells (Fig. 2a, Figure S3a, b). The models were trained on four different image types corresponding to staining conditions: H3K27ac only, CTCF only, Hoechst only, and all three channels combined. Model performance was evaluated using accuracy, precision, and area under the ROC curve (AUC) (Fig. 2b, Figure S3c). Most models achieved similar performance, except for the Hoechst-only model, which performed notably worse. The model trained on all three channels achieved the highest performance. Among single-channel models, the H3K27ac-only model performed closest to the all-channel model (accuracy: 0.996 vs. 0.994). This indicates that the H3K27ac channel is most informative for distinguishing VPA treatment, consistent with VPA’s role as a histone deacetylase inhibitor. The Hoechst-only model showed lower accuracy (0.896), suggesting that nuclear DNA staining alone is insufficient to distinguish epigenetic states. These results suggest that comparative model analysis across staining channels may help identify epigenetic modifications associated with specific biological processes (e.g., H3K27ac in VPA treatment).

### Nuclear periphery H3K27ac signal changes as image-based features in VPA-treated cells

Our machine learning model not only discriminates epigenetic states but also identifies contributing image features, enabling model interpretation. We applied Score-CAM [47], an enhanced class activation mapping (CAM) method [69], to highlight image regions relevant to model output. Visual inspection of representative Score-CAM heatmaps (Fig. 2c) revealed that VPA-treated cells exhibited globally elevated H3K27ac signal, along with distinct signal enrichment at the nuclear periphery. To validate this observation, we first generated average heatmaps for each condition by aligning individual Score-CAM heatmaps based on fitted ellipses (Fig. 2d, Figure S4a). These averaged heatmaps confirmed both the overall increase in H3K27ac signal in VPA-treated cells and the notable periphery-biased enrichment pattern.

We then quantified these differences through two metrics: the average pixel intensity of each heatmap, and the nuclear periphery enrichment calculated by dividing each nucleus into five concentric regions and measuring the ratio of Score-CAM intensity in the outermost region relative to the total nuclear intensity (Figure S4b; Methods). Quantitatively, VPA-treated cells exhibited significantly higher average intensities for H3K27ac (*p* = 3.37e-59) and modestly for CTCF (*p* = 1.34e-3) compared to untreated cells (Fig. 2e). Importantly, the nuclear periphery enrichment was also significantly elevated in VPA-treated cells across H3K27ac (*p* = 0.0) and CTCF (*p* = 0.0), supporting the hypothesis that peripheral signal enrichment is a characteristic epigenetic feature induced by VPA.

### Spatial reorganization of epigenetic features in VPA-treated cells

We further extracted punctate structures (“particles” or “spots”) from individual nuclei, referred to as nuclear puncta (Methods) and quantitatively analyzed their morphological and spatial features (Fig. 2f-h, **Figure S4c**). With the 120 nm imaging resolution, nuclear puncta observed in the H3K27ac and CTCF channels likely represent functional nuclear structures. H3K27ac puncta may correspond to active enhancers or other transcriptionally active regulatory regions, whereas CTCF puncta may represent with chromatin architectural anchors such as TAD boundaries or loop-forming elements.

Quantitative analysis revealed distinct trends across treatment conditions. VPA treatment increased the number of nuclear puncta for both H3K27ac (*p* = 0.0) and CTCF (*p* = 0.0), while their average area decreased (H3K27ac *p* = 3.93e-209; CTCF *p* = 0.0), suggesting that the increase in the number may reflect fragmentation into smaller domains. Signal intensity, particularly for H3K27ac, was markedly enhanced by VPA treatment (H3K27ac *p* = 7.23e-69; CTCF *p* = 6.51e-21), in line with its role as a histone deacetylase inhibitor. The shape of nuclear puncta also became more irregular in VPA-treated cells (H3K27ac *p* = 0.0; CTCF *p* = 2.12e-244), indicating greater structural variability or complexity (Fig. 2f).

To assess the spatial distribution of nuclear puncta, we divided each nucleus into five concentric regions (part1 to part5) from the innermost to the outermost layer. For each region, we calculated the puncta density as the number of segmented puncta per 250-pixel unit area (Methods) to compare the puncta enrichment across nuclear zones. In untreated cells, both H3K27ac and CTCF signals showed relatively uniform distribution across the nucleus with comparable puncta densities from part1 to part5. In contrast, VPA-treated cells exhibited a decline in puncta densities from the nuclear interior (part1) to the periphery (part5), indicating a shift of H3K27ac and CTCF signals along the radial direction (Fig. 2g). This trend was particularly pronounced for H3K27ac, consistent with the Score-CAM analysis highlighting H3K27ac change at the nuclear periphery (Fig. 2e). These findings suggest that VPA treatment not only alters the intensity of histone modifications but also redistributes their spatial organization within the nucleus.

The analysis of minimum distance between H3K27ac and CTCF puncta revealed that their spatial relationship was altered by VPA treatment (Fig. 2h). In VPA-treated cells, H3K27ac and CTCF puncta were more frequently located within short-range proximity (0–20 pixels) compared to untreated cells. This suggests a spatial reorganization of active chromatin regions relative to architectural chromatin boundaries. The observed reduction in their spatial separation may reflect a VPA-induced convergence of active regulatory elements toward domain boundaries, potentially due to increased chromatin accessibility or altered enhancer-promoter dynamics. These results imply that VPA not only modifies local histone acetylation but may also impact higher-order chromatin topology by modulating the relative positioning of functional chromatin elements.

In addition to the minimum-distance analysis, we quantified the spatial overlap between H3K27ac and CTCF puncta (**Figure S6c**) using the puncta overlap ratio metric (Methods). When using H3K27ac puncta as the reference, the overlap ratio increased from 0.210 in control cells to 0.269 in VPA-treated cells (*p* = 2.04e-165). When using CTCF puncta as the reference, the increase was more pronounced, rising from 0.070 to 0.174 (*p* = 0.0). These results suggest that VPA treatment enhances the spatial coincidence of H3K27ac and CTCF puncta, consistent with the reduced minimum-distance separation (Fig. 2h). Together, these findings further support that VPA induces a closer coupling between active regulatory elements and architectural chromatin sites.

Together, these results highlight distinct and quantifiable image-based features of nuclear organization associated with epigenetic states, suggesting peripheral enrichment of H3K27ac, increased puncta count and altered CTCF colocalization patterns as hallmarks of chromatin remodeling induced by VPA treatment. Our results demonstrate the feasibility of our proposed image-based epigenetic profiling approach for quantitatively characterizing nuclear architecture reorganization under pharmacological modulation.

### Sequencing analysis uncovers chromatin changes in lamina-associated domains in VPA-treated cells

**Fig. 3:**
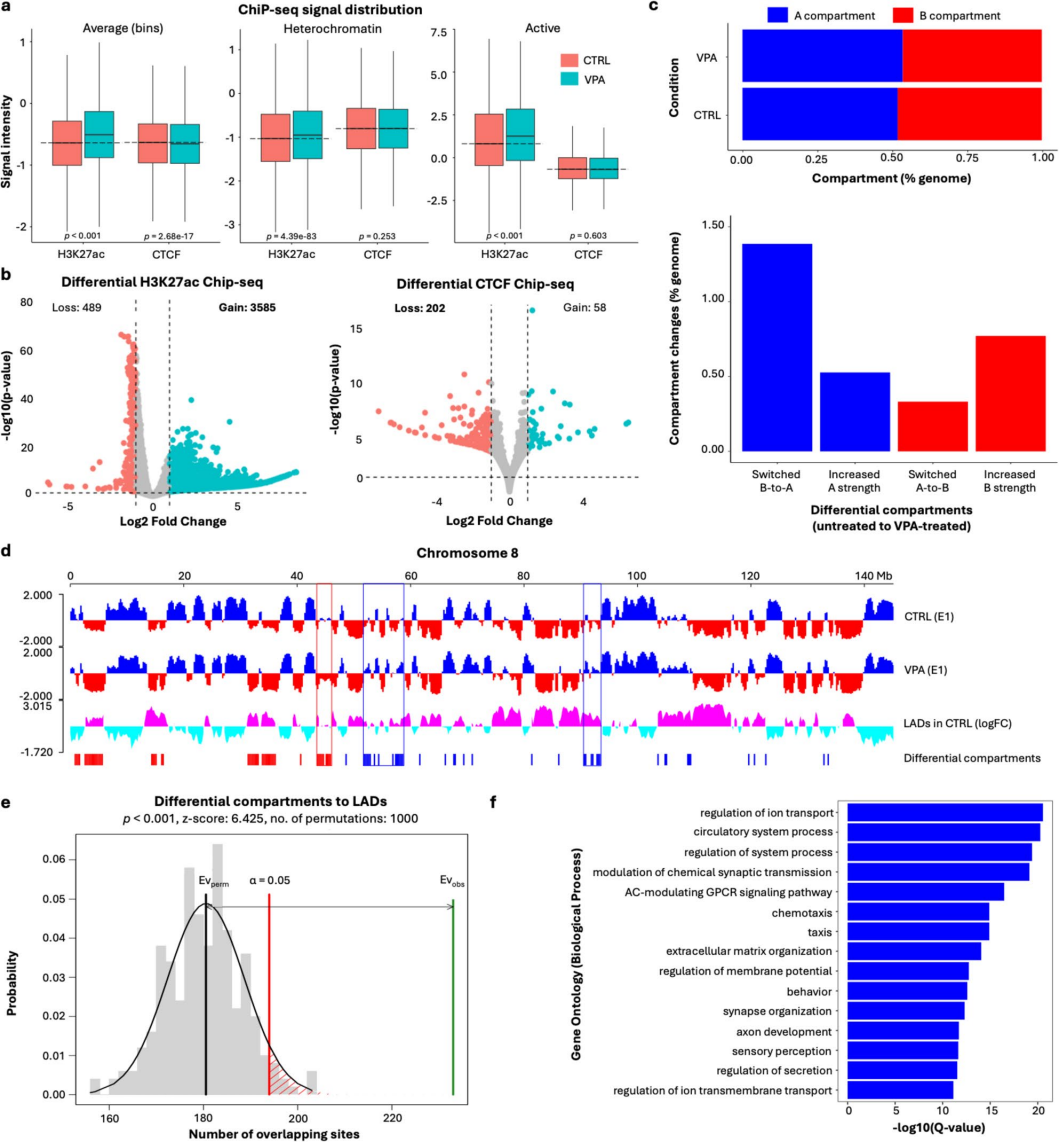
Sequencing-based epigenomic analysis on HEK293T cells. **a** Distribution of ChIP-seq signals: (left) average signals in 10 kb genomic bins, (middle) heterochromatin regions defined by the ChromHMM *Het* annotation; (right) active regions defined by ChromHMM annotations associated with transcriptionally active states (*Enh*, *Tss*). **b** Differential ChIP-seq peaks by DiffBind. Gained peaks describe peaks that are enriched in VPA-treated cells and lost peaks describe peaks that are enriched in control cells. **c** A/B compartments (top) and differential compartments (bottom) in untreated and VPA-treated cells. Differential compartments were defined as regions with first eigenvector value differences more than 0.5. **d** An example genome browser snapshot showing the distribution of A/B compartments and LADs in chromosome 8. A/B compartments are represented by the first eigenvector (E1) values from principal component analysis on Hi-C matrices. Positive values represent A compartments (blue), and negative values represent B compartments (red). LADs (magenta) and non-LADs (cyan) are represented by the log fold change (logFC) between Dam-LMNB1 expression and background methylation, from reported DamID data [67]. Boxes represent examples of differential compartments overlapped with LADs. **e** Overlap between differential compartments and LADs analyzed by permutation test using RegioneR [68]. The x-axis represents the number of overlapping sites, and y-axis represents the probability density, showing the estimated null distribution from 1000 permutations of randomly selected regions from differential compartments and LADs. The red vertical line indicates the significance threshold of *p* = 0.05. The green line denotes the observed number of overlapping regions (*p* < 0.001). **f** Gene Ontology enrichment of differentially expressed genes, ranked by FDR of Benjamini and Hochberg (BH) method (*q*-value) from top to bottom

At the nuclear periphery, the chromatin is known to associate with nuclear lamina, constituting lamina-associated domains (LADs) that are often heterochromatic. LADs interact with nuclear lamina through lamina-associated proteins such as Lamin A/C, Lamin B1 and Lamin B2 [70, 71]. Based on the nuclear periphery image features detected by Score-CAM and nuclear puncta analysis, we speculated that the epigenetic changes between VPA-treated and untreated cells may occur in LADs. To test this hypothesis, sequencing-based epigenomic analyses were performed by ChIP-seq, HiC and RNA-seq. We then combined these data with reported DamID data in HEK293 cells [67] to investigate whether the epigenetic change induced by VPA was enriched in LADs (Fig. 3). The overlap between genomic regions detected from different sequencing data was evaluated with *p*-value and *z*-scores by permutation test where positive z-scores represent strong overlap or enrichment.

ChIP-seq data showed significant increase of H3K27ac and slightly decreased CTCF read coverage across the entire genome (Fig. 3a). The genome-wide increase in H3K27ac ChIP-seq peaks correlates well with the elevated H3K27ac pixel intensity observed in imaging data (Fig. 2e). We also evaluated the potential enrichment of H3K27ac and CTCF at the heterochromatin and transcriptionally active chromatin regions defined by ChromHMM annotations in HEK293T cells. We observed a significant increase in H3K27ac levels following VPA treatment in both heterochromatin and active chromatin regions (heterochromatin, *p* = 4.39e-83; active, *p* < 0.001) (Fig. 3a). The magnitude of the nuclear periphery signal change observed in the imaging results was more pronounced (Fig. 2e) compared to relatively subtle differences detected by ChIP-seq (Fig. 3a). This may arise from the different focus of the analysis; while the imaging results in Fig. 2e directly focus on nuclear periphery, the ChIP-seq results in Fig. 3a use the heterochromatin and active regions, which may contain regions other than nuclear periphery. Meanwhile, differential peak analysis by DiffBind found a significant gain of H3K27ac and loss of CTCF peaks (Fig. 3b).

Hi-C data showed that, by VPA treatment, the A compartments that represent open/active chromatin regions were increased while the B compartments that represent closed/repressive chromatin regions were decreased (Fig. 3**cd**). The B compartments in both conditions were highly correlated with LADs (Pearson’s correlation coefficient, *r* = 0.7), which is consistent with previous studies in other cell types [70, 71].

Hi-C differential compartments significantly overlapped with LADs (*p* < 0.001, *z*-score = 7.5) (Fig. 3e). Specifically, the B-to-A switched regions (i.e., the regions whose A/B compartment assignments were changed from B to A by VPA treatment) were more enriched in LADs (*p* < 0.001, *z*-score = 5.2) compared with the A-to-B switched regions (*p* = 0.3646, *z*-score = 0.6). Interestingly, the changes in TAD boundaries (i.e., TAD boundaries gained or lost by VPA treatment) also significantly occurred in LADs (*p* < 0.001, *z*-score = 4.6) while the changes of H3K27ac and CTCF binding sites were depleted in LADs (*p* < 0.001, *z*-score = −74.5 and − 18.7 respectively). These results validated that LADs could be a hotspot of the epigenetic change induced by VPA treatment, which was characterized by the B-to-A compartment switch.

 To elucidate the gene regulatory changes by VPA treatment, we analyzed differentially expressed genes (DEGs) between VPA-treated and untreated cells using RNA-seq. 2037 genes were up-regulated and 86 genes were down-regulated following VPA treatment (log_2_ fold change ≥ 1 or ≤ − 1, FDR-adjusted *q* < 0.05) (Table S1). Gene Ontology analysis showed that these DEGs were significantly enriched in histone deacetylase activities and other biological process known to be associated with VPA (Fig. 3f) that were similarly observed in previous studies on VPA-treated cells, such as regulation of ion transport [72], modulation of chemical synaptic transmission [72], chemotaxis [73, 74], extracellular matrix organization [73–76] and synapse organization [75–77].

The integration of ChIP-seq and RNA-seq data showed a significant overlap between DEGs and differential H3K27ac peaks (*p* < 0.001, z-score = 9.2) (Figure S5a). Specifically, down-regulated genes were enriched in lost peaks (peaks only in untreated cells) (*p* < 0.001, z-score = 20.5) (Figure S5b) while up-regulated genes were enriched in gained peaks (peaks only in VPA-treated cells) (*p* < 0.001, z-score = 9.6) (Figure S5c). These results indicate a significant correlation between gene regulation and the epigenetic changes of H3K27ac status.

Despite the lack of significant overlap between the LADs and DEGs (z-score = −7.9), approximately 30% of DEGs were localized in LADs, with 37 and 3 up-regulated genes in B-to-A and A-to-B switched compartments, respectively. This indicates that LADs were not completely restricted to transcriptional repression, which are in line with previous reports that LADs may also contribute to gene activation necessary for chromatin remodeling [78, 79]. For example, several chromatin-modifying genes were up-regulated in the B-to-A switched compartments overlapped with LADs, such as *SYNE1* (log_2_ fold change = 1.2) and *SP110* (log_2_ fold change = 1.4). *SYNE1* encodes nesprin-1, a component of the nuclear envelope located in the nuclear periphery. The protein is located on the outer nuclear membrane and links the nuclear lamina to the cytoskeleton as a part of the linker of nucleoskeleton and cytoskeleton (LINC) complex [80]. Nesprin-1 interacts with SUN proteins, another component of the LINC complex, which connect to the inner nuclear membrane through interactions with Lamin A and chromatin [80, 81]. Disruption of the LINC complex is reported to alter the ratio of euchromatin and heterochromatin [82].

**Fig. 4:**
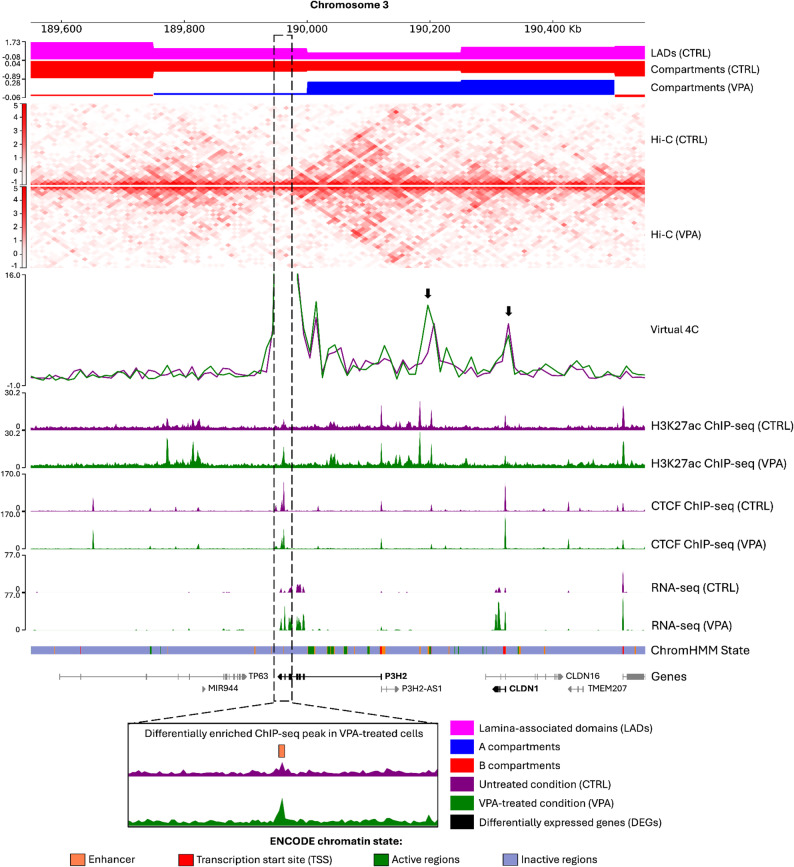
Up-regulation of *P3H2* and *CLDN1* in B-to-A switched compartments and LADs. Our epigenomic data were shown with the reported LAD data and the chromatin state annotations by ChromHMM on HEK293T cells from ENCODE. The up-regulation of *P3H2* and *CLDN1* was concomitant with a differential H3K27ac peak in a putative intronic enhancer interacting with a distal upstream enhancer and a promoter of these genes (black arrows). Up-regulated genes are indicated in bold. The box with black dashed lines highlights the differential H3K27ac peak at the *P3H2* locus (log2 fold change = 1.5, FDR < 0.05). The virtual 4 C track represents the Hi-C interaction with the differential H3K27ac peak as a viewpoint

*P3H2* (log_2_ fold change = 1.2) and *CLDN1* (log_2_ fold change = 2.6) were also up-regulated in the B-to-A switched compartments overlapped with LADs (Fig. 4). The *P3H2* gene encodes the prolyl 3-hydroxylase 2 protein involved in collagen assembly, while *CLDN1* encodes claudin-1, which functions in tight junction assembly. Both genes have been reported to be regulated by the common transcription factor p63 associated with breast cancer progression [83–85]. The up-regulation of *P3H2* was concomitant with a differential H3K27ac peak at the intronic region gained in VPA-treated cells (log_2_ fold change = 1.5) (Fig. 4). This H3K27ac peak was mapped to a putative enhancer of *P3H2* annotated by ChromHMM and a candidate cis-regulatory elements in HEK293T cells from ENCODE (ENCODE accessions: ENCSR770TKR and ENCSR751GSA, respectively) [86] (Fig. 4). This locus also had chromatin interactions with the distal upstream enhancer of *P3H2* and the promoter of *CLDN1* in our Hi-C data. Thus, the activation of the enhancer by VPA treatment might lead to the up-regulation of these genes. Motif analysis of differential CTCF peaks revealed significant enrichment of canonical CTCF motif and potential co-factors, as expected (Table S2). Meanwhile, differential H3K27ac peaks were significantly enriched with motifs associated with developmental transcription factors (Table S2), suggesting a prominent role for these factors in regions with altered enhancer activity.

### Image-based analysis of RTT cells reveals epigenetic signal changes

To further evaluate the generalizability of our framework, we analyzed a disease-relevant model of RTT. The iPS cells were derived from three RTT patient lines: HPS3042, harboring a mutation in MBD; and HPS3049 and HPS3084, both carrying mutations in TRD. We applied the same image-based profiling as performed in the VPA case to characterize epigenetic changes associated with *MECP2* deficiency.

**Fig. 5:**
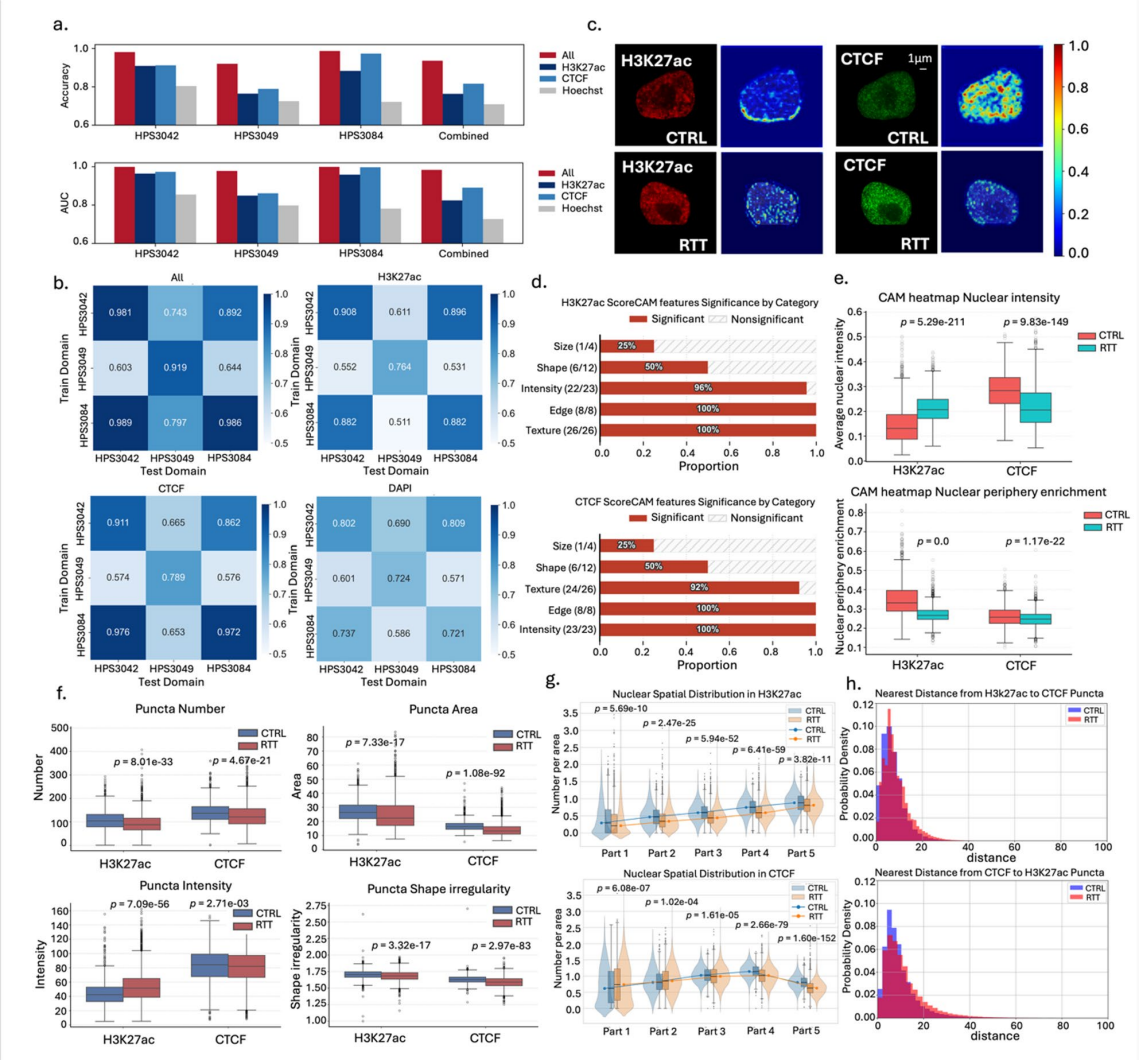
Image-based epigenetic analysis in RTT cells. **a **Classification performance of the same CNN model applied to RTT and control cells (CTRL). Results are shown for the model trained separately on each mutation line, and that trained across all lines. **b** Cross-domain generalization performance of models trained on individual lines. Heatmaps show classification accuracy when the model trained on one line (row) is applied to the other line (column), for each staining channel. Diagonal values represent within-domain performance, while off-diagonal values indicate cross-domain transferability. **c** Representative Score-CAM heatmaps. Examples for CTRL and RTT cells (HPS3042) under H3K27ac and CTCF staining. Red regions indicate areas most strongly contributing to classification. **d** Quantitative analysis of Score-CAM heatmaps using the standard image features (Methods). For each feature category (the number of features shown in parenthesis), the bar plot represents the fraction of features with significant difference between RTT and CTRL cells. **e** Quantitative analysis of Score-CAM heatmap nuclear intensity and nuclear periphery enrichment. **f–h** Quantitative analysis of nuclear puncta, including number, area, intensity, shape, spatial distribution, and colocalization

### Machine learning models to discriminate RTT cells and control cells

We used the same model architecture (Fig. 2a) and training strategy as in the VPA case to discriminate between RTT and control cells. To investigate the impact of *MECP2* mutations, we analyzed the data from two perspectives: staining conditions and mutated domains.

Among staining conditions, the Hoechst-only model performed the worst, indicating that DNA staining alone provides limited epigenetic information, as in the VPA case. The all-channel model performed best, confirming that combining multiple markers improves classification. Notably, CTCF yielded higher accuracy than H3K27ac (Fig. 5a), suggesting that *MECP2* mutations may lead to more consistent alterations in chromatin architecture than in histone acetylation patterns.

Models trained separately on each mutation line showed notable variability. HPS3084 performed similarly to HPS3042 and substantially better than HPS3049, despite both HPS3084 and HPS3049 sharing TRD mutations. This suggests that not all TRD mutations exert equal effects on chromatin architecture, possibly due to differences in the mutation positions or patient-specific genetic context. To further examine the consistency between domains, we performed cross-domain validation by training models on one mutation line and testing on the others (Fig. 5b). Results revealed remarkably strong generalizability between HPS3084 and HPS3042, indicating that a model trained on either line can classify the other with substantial accuracy. This cross-line compatibility is noteworthy, suggesting that these two lines share key epigenetic features that allow effective transfer of predictive signals. In contrast, models trained on HPS3049 showed limited ability to transfer, underscoring its distinct epigenetic profile.

#### Nuclear periphery H3K27ac signal changes in RTT cells

We visualized the Score-CAM heatmaps to identify the image regions contributed to classification (Fig. 5c) and quantified the standard image features [49] to analyze Score-CAM heatmaps (Methods). We grouped these image features into five categories: “Size”, “Shape”, “Intensity”, “Edge”, “Texture”, then showed the fraction of features with significant differences between RTT and control cells (Fig. 5d). The most prominent differences were observed in the “Intensity”, “Texture” and “Edge” categories. In contrast, shape and size metrics showed minimal change.

In the nuclear intensity and nuclear periphery enrichment analyses (Fig. 5e), RTT cells showed increased nuclear intensity (*p* = 5.29e-211) and decreased nuclear periphery enrichment (*p* = 0.0) in the H3K27ac Score-CAM heatmaps, indicating a redistribution of signal toward the nuclear interior. In the CTCF Score-CAM heatmaps, both nuclear intensity (*p* = 9.83e-149) and nuclear periphery enrichment (*p* = 1.17e-22) were reduced in RTT cells, suggesting less contribution to classification.

### Altered Spatial distribution of epigenetic features in RTT cells

The nuclear puncta analysis (Fig. 5f) revealed a reduction in puncta number and area in RTT cells compared to control cells, for both H3K27ac (puncta number, *p* = 8.01e-33; puncta are, *p* = 7.33e-17) and CTCF (puncta number, *p* = 4.67e-21; puncta area *p* = 1.08e-92). Notably, H3K27ac puncta exhibited increased average intensity in RTT cells (*p* = 7.09e-56). In contrast, CTCF puncta intensity slightly decreased (*p* = 2.71e-03). Puncta shape became more irregular in RTT cells (H3K27ac *p* = 3.32e-17; CTCF *p* = 2.97e-83).

In the nuclear spatial distribution analysis (Fig. 5g), H3K27ac puncta density in control cells showed an outward gradient, increasing from center to periphery. RTT cells maintained the same gradient direction but showed globally lower densities across all nuclear regions, indicating a reduction in active enhancer–associated puncta. For CTCF, the control pattern was more uniform, with the highest density in mid-to-outer nuclear zones. RTT cells exhibited a gradual interior-to-periphery increase, suggesting a relocation of architectural anchors toward the nuclear edge. These spatial redistribution patterns suggest *MECP2* loss disrupts both enhancer activity and chromatin domain structures.

The minimum-distance analysis revealed that in RTT cells, H3K27ac and CTCF puncta were less frequently located within the 0–10 pixel range and more frequently located within the 10–20 pixel range compared to control cells, indicating an overall increase in spatial separation rather than closer proximity (Fig. 5h). The overlap ratio metric also revealed reduced spatial coincidence between H3K27ac and CTCF puncta in RTT cells (Figure S7c). Using H3K27ac as the reference, the overlap ratio decreased from 0.318 in control cells to 0.224 in RTT cells (*p* = 4.61e-197). Using CTCF as the reference, the overlap ratio decreased from 0.266 to 0.174 (*p* = 4.32e-184). The increased minimum distance and reduced overlap between H3K27ac and CTCF puncta in RTT cells indicate greater spatial dispersion between active regulatory elements and architectural boundaries. Notably, MECP2 loss is known to weaken chromatin compaction and TAD boundary insulation, which can lead to a loosening of enhancer–boundary coupling [87]. Thus, the puncta pattern observed in RTT cells may reflect such a decoupling between H3K27ac and CTCF marks.

Together, these results demonstrate that RTT is associated with specific and quantifiable alterations in nuclear epigenetic architecture, including reduced enhancer density, relocalization of CTCF-bound elements, and decoupling between H3K27ac and CTCF marks. These signatures offer image-based features for *MECP2* mutation and illustrate the capacity of our framework to reveal spatially resolved chromatin remodeling in disease contexts.

#### ChIP-seq analysis revealed chromatin changes in RTT cells consistent with image-based findings

**Fig. 6:**
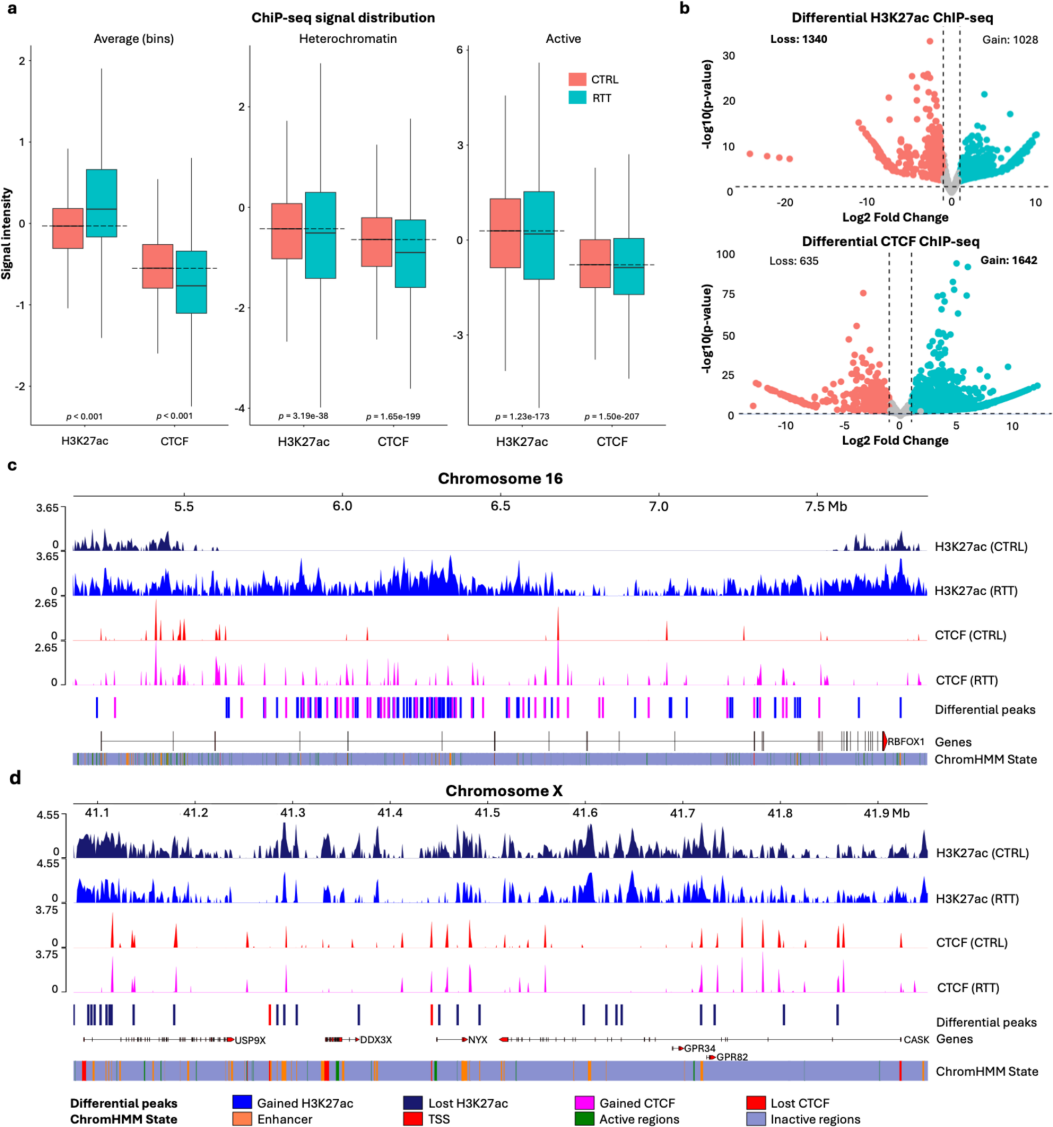
Sequencing-based epigenomic analysis on RTT iPS cells. **a** Distribution of ChIP-seq signals: (left) average signals across 10 kb genomic bins; (middle) heterochromatin regions defined by the ChromHMM *Het* annotation; (right) active regions defined by ChromHMM annotations associated with transcriptionally active states (*Enh*, *Tss*). **b** Differential ChIP-seq peaks in RTT cells compared to control cells (CTRL) detected by DiffBind. **c** Enrichment of H3K27ac and CTCF signals within the *RBFOX1* gene body in RTT cells with overlapping putative enhancers. Blue and magenta in the ‘Differential peaks’ track indicates gained H3K27ac and CTCF peaks, respectively. **d** Significant loss of H3K27ac and CTCF peaks near several X-linked genes. Dark blue and red in the ‘Differential peaks’ track indicates lost H3K27ac and CTCF peaks, respectively

The image-based analyses showed that RTT led to increased H3K27ac and decreased CTCF Score-CAM intensities in the entire nucleus (Fig. 5e). Meanwhile, the Score-CAM intensities decreased at the nuclear periphery for both markers (Fig. 5e). ChIP-seq analysis showed increased H3K27ac, and decreased CTCF intensities at the whole-genome scale, which match well with the image-based findings (Fig. 6a). In ChromHMM-defined heterochromatin and active regions of iPS cells, both H3K27ac and CTCF signals were significantly reduced (Fig. 6a). Since the nuclear periphery comprises inactive heterochromatin and active chromatin regions, these ChIP-seq changes may correspond to the nuclear periphery changes detected by image-based analysis.

Differential ChIP-seq analysis by DiffBind revealed gained and lost peaks in RTT cells compared with control cells (Fig. 6b). Despite the global increase of H3K27ac intensity (Fig. 6a), lost peaks were more frequent than gained peaks. Closer inspection revealed that a subset of regions exhibited markedly increased H3K27ac enrichment, likely corresponding to active enhancers. An example is the *RBFOX1* gene body (Fig. 6c). RBFOX1 has known associations with MeCP2 and RTT [88]. The RBFOX1 protein functions in splicing regulation critical for synaptic transmission in the brain. The MECP2 protein binds to the promoter region of *RBFOX1* to regulate the assembly of Rbfox/LASR protein complexes; *MECP2* mutation disrupts the complex formation, possibly affecting the neuronal disorder in RTT. For other examples, *H3C3*, *KDM7A*, *CNTN4*, and *CNTN6* loci gained H3K27ac and CTCF peaks, while *BCOR* and *CASK* showed significant loss of CTCF peaks, which have known associations with MeCP2 and RTT [89–92].

 Several X-linked genes including *CASK*, *GPR34*, *GPR82*, *NYX*, *DDX3X*, and *USP9X*, exhibited combined loss of H3K27ac and CTCF peaks (Fig. 6d), which are critical for brain function [93]. These findings indicate that *MECP2* mutation not only alters histone acetylation but also redistributes CTCF binding, thereby reshaping key gene networks involved in neurodevelopment and epigenetic regulation. As expected, motif analysis on differential CTCF ChIP-seq peaks revealed significant enrichment of the canonical CTCF motif, as well as enrichment of motifs for potential co-factors (**Table S3**).

Overall, our results demonstrate that image-based predictions effectively capture changes in chromatin markers such as H3K27ac and CTCF, not only in VPA-treated cells but also in RTT cells. Notably, the image-based approach identified chromatin features not readily detectable by ChIP-seq alone and motivated our subsequent sequencing analyses. Imaging preserves morphological changes, subnuclear positioning and spatial distribution from puncta-based analysis, such as nuclear periphery enrichment patterns of H3K27ac and CTCF, that bulk sequencing cannot capture due to chromatin fragmentation and the averaging of signals across heterogenous cell populations [94]. While sequencing offers the genome-wide scale, it requires substantial downstream annotation to infer structural or spatial context. For instance, our ChIP-seq data supported the molecular changes suggested by the imaging results, including the enrichment pattern of H3K27ac and CTCT within heterochromatin and active regions, by reference to external ChromHMM annotations. In addition, the DamID-defined LAD alterations in HEK293T cells would not have been evident from the imaging data alone. Thus, the two approaches are highly complementary, where imaging identifies architectural alterations, and sequencing provides molecular detail that extends and strengthens these observations.

#### Impact of super-resolution imaging on epigenetic profiling performance

**Fig. 7:**
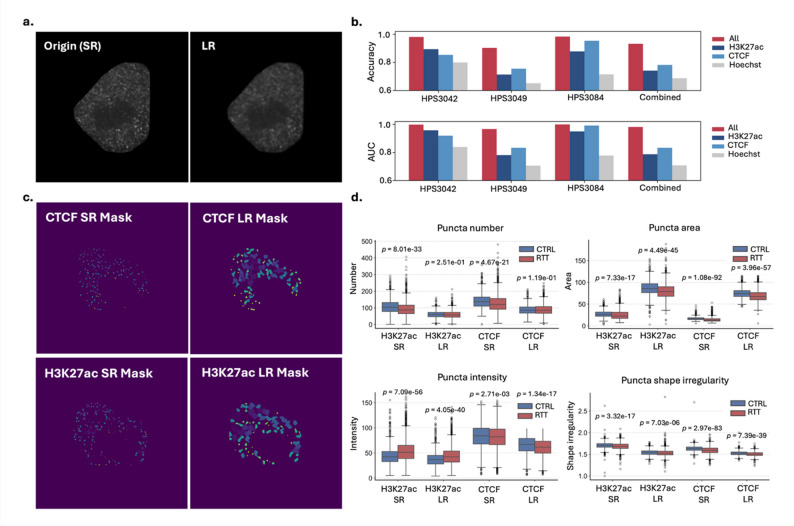
Analysis of simulated low-resolution image in RTT and control cells. **a** Comparison between original super-resolution (SR) images and simulated low-resolution (LR) images. **b** Classification performance of the same CNN model applied to RTT and control cells using LR data. Results are shown for models trained separately on each mutation line as well as for a combined model trained across all lines. **c** Examples of puncta segmentation in LR images. **d** Quantitative analysis of nuclear puncta properties in LR images compared to SR images

To assess the necessity of super-resolution (SR) imaging for accurate image-based epigenetic profiling, we simulated low-resolution (LR) images corresponding to a typical confocal microscopy by applying Gaussian blurring to the original SR images of RTT and control cells (Methods). A visual comparison between SR and simulated LR images is shown in Fig. 7a. Using the same pipeline as for SR data, we trained classification models and performed quantitative analyses of nuclear puncta features.

In classification tasks, models trained on LR images consistently underperformed compared to those trained on SR images across all staining conditions and domains (Fig. 7b). This performance gap suggests that SR imaging captures more discriminative features relevant to epigenetic state differences. While the overall trends were largely preserved between SR and LR data such as the relative classification performance across domains (e.g., HPS3042 and HPS3084 showed higher accuracy than HPS3049) and within-domain staining order (e.g., All combined > CTCF > H3K27ac > Hoechst), some divergences were observed. For example, in HPS3042, classification performance for CTCF dropped below that of H3K27ac in the LR condition, being opposite to the SR condition. These exceptions highlight that while LR images may preserve broad epigenetic trends, SR imaging remains essential to capture finer features for accurate interpretation.

We applied our nuclear puncta segmentation pipeline to the LR images (Fig. 7c) and extracted quantitative features including puncta number, area, intensity, and shape irregularity (Fig. 7d). By comparing SR and LR data, we found that SR data captures a greater number of puncta with smaller areas, suggesting the collapse of puncta in the LR data. Notably, the difference of H3K27ac puncta numbers between RTT and control cells detected in the SR data (*p* = 8.01e-33), became less statistically significant (*p* = 0.251), further supporting the ability of SR imaging to capture epigenetic changes between biological conditions.

Together, these results demonstrate that SRM is essential for high-fidelity epigenetic profiling, enabling to capture subtle nuclear features that would be lost in LR imaging.

## Discussion

The low throughput of typical SRM has impeded its applications with machine learning in image-based epigenetic profiling. We have overcome the limitation by employing a high-speed SRM that enabled the fast acquisition of super-resolution cell images. With the large dataset of thousands of single-cell super-resolution images, we showed an application of deep learning for cell classification and the discovery of image features contributing to classification. Using VPA-treated HEK293T cells and RTT iPS cells as models, our deep learning models accurately discriminated epigenetic states and identified key image features. Combined with the nuclear puncta analysis, we detected the nuclear periphery as a hotspot of epigenetic changes, and the nuclear spatial distribution and colocalization of H3K27ac and CTCF foci characteristic under each perturbation. The sequencing-based epigenomic analyses further revealed the epigenetic changes at LADs, being consistent with the image-based findings.

In recent years, image-driven epigenetic studies have expanded rapidly. Sequencing-based methods such as ChIP-seq, Hi-C, and RNA-seq provide genome-wide profiles of chromatin modifications but lack spatial resolution [95], while SRM enables nanoscale visualization of chromatin but has been limited by low throughput [96]. Prior approaches, such as the MIEL pipeline [97], used histone mark images (e.g., H3K9me3, H3K27ac) and texture features with classical classifiers to distinguish cells treated with different compounds. Cell painting and high-content screening have also been applied for drug phenotyping [98], but these mainly rely on morphological or texture descriptors and lack chromatin-level interpretation.

More recently, imaging has been integrated with omics approaches, including transcriptomics [99–101], genomics [102–104], and in situ genome sequencing [105, 106] to better resolve cellular organization and link imaging features to underlying genomic regions at single-cell resolution. multiplexed fluorescence in situ hybridization (FISH) techniques, such as MERFISH [100, 102] and DNA seqFISH+ [103, 104], enable imaging of RNA transcripts or genomic loci at single-molecule resolution. In situ genomic sequencing (IGS) allows simultaneous sequencing of genomic DNA in intact cells, providing information on chromatin domains and chromosome positioning [105, 106]. These approaches facilitate inference of chromatin organization and higher-order nuclear architecture, offering insights into epigenetic regulation. However, the effective spatial resolution of these approaches is implementation-dependent: standard FISH is diffraction-limited (though FISH spots can be localized with sub-pixel precision in super-resolution variants), and IGS typically operates at tens-to-hundreds of nanometers but can be improved by expansion microscopy; nevertheless, resolving fine subnuclear structures remains challenging compared with direct nanometer-scale SRM.

In contrast, SRM provides nanometer-scale visualization of chromatin features, such as H3K27ac-marked active regions and CTCF-bound architectural sites. Combined with deep learning–based image analysis, it enables automated segmentation, quantification of nuclear puncta, detection of subtle morphological changes, and classification of cell states, capturing heterogeneity that bulk sequencing or spatial-omics cannot resolve. Moreover, super-resolution imaging preserves native nuclear architecture, allowing precise mapping of epigenetic marks to subnuclear compartments, such as the nuclear periphery. Integrating super-resolution microscopy with computational analysis thus complements genome-wide assays like ChIP-seq, Hi-C, or RNA-seq, linking molecular changes to spatial nuclear organization and cellular identity.

We present an image-based framework that combines high-speed SRM, deep learning classification, Score-CAM interpretation, nuclear puncta segmentation, with the further sequencing analyses by ChIP-seq, Hi-C, and RNA-seq. This pipeline overcomes throughput limitations of SRM, enables pixel-level single-cell analysis, and provides interpretable metrics of chromatin puncta abundance, morphology, distribution, and proximity. Biologically, it reveals perinuclear H3K27ac enrichment in VPA-treated cells and links these image features to LAD-associated B-to-A compartment switching. Compared with earlier imaging approaches that relied on averaged texture features or lacked sequencing validation, our work provides higher-resolution, interpretable image-based signatures and establishes a direct link between imaging features and chromatin regulatory mechanisms.

Our deep learning approach elucidated the importance of nuclear periphery as a key feature for epigenetic changes using SRM data alone. The detected nuclear periphery image features are partly supported by previous experimental studies on various cell types and perturbations. In many cell types, heterochromatin is known to localize preferentially to the nuclear periphery through the interaction with lamina-associated proteins including lamin (LMNA/B), emerin (EMD), and heterochromatin proteins (HP1) [107, 108].

VPA has been reported to induce chromatin decompaction through histone modification, which in turn affects the nuclear integrity maintained by heterochromatin [7, 107]. For example, in normoglycemic hepatocyte cells, Felisbino et al. [107] reported the increase of H3K9ac and the shift of its localization towards the nuclear periphery after VPA treatment, as well as the dissociation of HP1 from heterochromatin to the cytoplasm. Enriched histone acetylation around the nuclear periphery were also reported in colon adenocarcinoma HT29 [109], HeLa [110] and human fibroblast cells [111] after treatment with Trichostatin A, another HDAC inhibitor. To the best of our knowledge, the present study is the first report on the effect of VPA on the localization of H3K27ac at the nuclear periphery in HEK293T cells. In addition, our study is the first to show the HDAC inhibitor-induced nuclear reorganization through both image-based and sequencing-based analyses.

MECP2’s involvement in RTT has been extensively investigated through imaging and sequencing individually, yet studies combining both approaches are limited. ChIP-seq analyses have consistently reported increased histone acetylation, including elevated H3K27ac in MECP2-deficient or RTT cells, particularly at highly methylated long genes [112–114]. The CTCF dynamics in RTT have been understudied, yet Kernohan et al. [115] showed that MECP2 loss increases nucleosome occupancy at CTCF sites and reduces CTCF binding, consistent with our observations. Meanwhile, fluorescence studies [116, 117] demonstrated that RTT-associated MECP2 mutations impair heterochromatin condensate formation via liquid–liquid phase separation, potentially altering the spatial segregation of chromatin domains and distribution of chromatin-bound factors such as CTCF. However, detailed imaging-based descriptions of CTCF and H3K27ac nuclear puncta have remained scarce. Together, our imaging and sequencing results provide a more holistic view of chromatin alterations in RTT.

To further clarify whether the nuclear-periphery features highlighted by Score-CAM originate from genuine epigenetic reorganization or from nuclear morphological cues, we performed additional analyses on the original SRM images across H3K27ac, CTCF, and Hoechst channels (Figure S6a-b, Figure S7a-b). For the CTRL–VPA comparison, both H3K27ac and CTCF raw images showed significant increases in nuclear intensity and periphery enrichment in VPA-treated cells (e.g., H3K27ac intensity *p* = 0.0; periphery *p* = 6.05e-3; CTCF intensity *p* = 2.39e-238; periphery *p* = 5.82e-41), consistent with the Score-CAM-derived importance maps. These results confirm that the periphery-associated Score-CAM-derived features for H3K27ac and CTCF reflect spatial changes in epigenetic signals, rather than nuclear shape or segmentation artifacts. Notably, while similar trends in nuclear intensity and periphery enrichment can be detected from raw images, the Score-CAM maps provide a complementary representation by localizing the image regions that contribute most strongly to classification. This spatial attribution is particularly valuable in the context of single-cell SRM data, where signal heterogeneity and imaging noise can obscure subtle but consistent spatial patterns when assessed using global intensity metrics alone. Thus, the deep learning framework does not replace conventional image-based measurements but enables a more localized and interpretable extraction of chromatin-associated features that are most relevant for distinguishing epigenetic states. In contrast, the Hoechst-only model, despite achieving a high AUC, showed strong nuclear-periphery activation in Score-CAM heatmaps that was not supported by the raw Hoechst images. In the CTRL–VPA comparison, the raw images exhibited opposite periphery enrichment (CTRL > VPA, *p* = 8.04e-74), indicating that the Score-CAM signal does not reflect the biological distribution of DNA. In RTT cells, the Score-CAM maps again showed marked periphery activation (*p* = 1.65e-195), whereas the raw Hoechst images showed no significant periphery enrichment (*p* = 0.0663). These discrepancies demonstrate that Hoechst-derived Score-CAM activation primarily arises from global nuclear morphological cues, such as chromatin condensation or nuclear contour changes, rather than DNA accumulation at the nuclear periphery.

Our method is based on 2D super-resolution imaging, which carries inherent limitations. Without z-axis information, the analysis cannot resolve volumetric chromatin organization, vertical layering of nuclear structures, or true 3D spatial relationships between chromatin domains. Measurements such as puncta morphology, spatial distribution, and perinuclear positioning are therefore confined to a single imaging plane. Chromatin architecture is fundamentally three-dimensional, and extending the framework to volumetric imaging would help address these constraints. 

A limitation of our analysis is the absence of internal nuclear landmarks (e.g., nuclear speckles), which limits the ability to assess relocalization of chromatin puncta relative to specific subnuclear structures. As a result, the analysis primarily captures changes associated with the nuclear periphery, where boundaries are well defined. Incorporating nuclear speckle or other subnuclear markers in future work would allow a more complete characterization of spatial chromatin repositioning.

Our study is limited by the lack of ability of current sequencing datasets to distinguish between active and inactive regions at the nuclear periphery. Although imaging showed altered H3K27ac and CTCF enrichment at the periphery, available sequencing resources cannot determine whether these changes occur in lamina-associated (inactive) or nuclear pore-associated (active) subdomains. Genome-wide nucleoporin-chromatin maps (e.g., Nup93 or Nup153 ChIP/DamID) are not available for HEK293T and RTT iPS cells, and datasets from other cell types are not suitable for reliable comparison. Consequently, our analysis focused on peripheral features supported by existing data, including DamID-defined LADs from HEK293T cells, and ChromHMM annotations available for both cell types. Future work incorporating lamina- and nucleoporin-based profiling in the relevant cell types would be valuable for distinguishing active versus inactive peripheral subcompartments.

## Conclusions

Overall, our study has demonstrated the image-based epigenetic profiling combining high-speed SRM and machine learning as a useful tool for cell classification and image-based feature discovery with the verification by sequencing-based analysis. As a future direction, this strategy will be applied to various biological processes involving epigenetic alterations such as development and disease, possibly contributing to image-based diagnosis.

## Supplementary Information

Supplementary Material 1

Supplementary Material 2

## Data Availability

The image data are available at Zenodo ([10.5281/zenodo.10032842](10.5281/zenodo.10032842)). The codes for image data analysis are available at Github ([https://www.github.com/dddogwang/disease_epigenome]). The sequencing data are available in NCBI GEO with the accession numbers GSE246399 for VPA treatment on HEK293T cells ([https://www.ncbi.nlm.nih.gov/geo/query/acc.cgi?acc=GSE246399](https://www.ncbi.nlm.nih.gov/geo/query/acc.cgi?acc=GSE246399)) and GSE308264 for RTT iPS cells ([https://www.ncbi.nlm.nih.gov/geo/query/acc.cgi?acc=GSE308264](https://www.ncbi.nlm.nih.gov/geo/query/acc.cgi?acc=GSE308264)).

## References

[1] Misteli T. The self-organizing genome: principles of genome architecture and function. Cell. 2020;183:28–45. 32976797 10.1016/j.cell.2020.09.014 PMC7541718

[2] Schaeffer M, Nollmann M. (2023) Contributions of 3D chromatin structure to cell-type-specific gene regulation. Curr Opin Genet Dev, 79. 10.1016/j.gde.2023.102032 36893484

[3] Jha RK, Levens D, Kouzine F. Mechanical determinants of chromatin topology and gene expression. Nucleus. 2022;13:94–115. 35220881 10.1080/19491034.2022.2038868 PMC8890386

[4] Venkatachalapathy S, Jokhun DS, Andhari M, Shivashankar GV. (2021) Single cell imaging-based chromatin biomarkers for tumor progression. Sci Rep, 11. 10.1038/s41598-021-02441-6 PMC8630115 34845273

[5] Wang M, Sunkel BD, Ray WC, Stanton BZ. (2022) Chromatin structure in cancer. BMC Mol Cell Biol, 23. 10.1186/s12860-022-00433-6 PMC9331575 35902807

[6] Lebeau B, Jangal M, Zhao T, Wong CK, Wong N, Cañedo EC, Hebert S, Aguilar-Mahecha A, Chabot C, Buchanan M et al. (2022) 3D chromatin remodeling potentiates transcriptional programs driving cell invasion. Proc Natl Acad Sci U S A, 119. 10.1073/pnas.2203452119 PMC9457068 36037342

[7] Carollo PS, Barra V. (2023) Chromatin epigenetics and nuclear lamina keep the nucleus in shape: examples from natural and accelerated aging. Biol Cell, 115. 10.1111/boc.202200023 36117150

[8] Stepanov AI, Besedovskaia ZV, Moshareva MA, Lukyanov KA, Putlyaeva LV. (2022) Studying chromatin epigenetics with fluorescence microscopy. Int J Mol Sci, 23. 10.3390/ijms23168988 PMC9409072 36012253

[9] Neganova ME, Klochkov SG, Aleksandrova YR, Aliev G. Histone modifications in epigenetic regulation of cancer: perspectives and achieved progress. Semin Cancer Biol. 2022;83:452–71. 32814115 10.1016/j.semcancer.2020.07.015

[10] Milon BC, Cheng H, Tselebrovsky MV, Lavrov SA, Nenasheva VV, Mikhaleva EA, et al. Role of histone deacetylases in gene regulation at nuclear lamina. PLoS One. 2012. 10.1371/journal.pone.0049692. 23226217 10.1371/journal.pone.0049692 PMC3511463

[11] Dirks RAM, Stunnenberg HG, Marks H. (2016) Genome-wide epigenomic profiling for biomarker discovery. Clin Epigenetics, 8. 10.1186/s13148-016-0284-4 PMC5117701 27895806

[12] Eagen KP. Principles of chromosome architecture revealed by Hi-C. Trends Biochem Sci. 2018;43:469–78. 29685368 10.1016/j.tibs.2018.03.006 PMC6028237

[13] Lieberman-Aiden E, Van Berkum NL, Williams L, Imakaev M, Ragoczy T, Telling A, et al. Comprehensive mapping of long-range interactions reveals folding principles of the human genome. Science. 2009;326:289–93. 19815776 10.1126/science.1181369 PMC2858594

[14] Rao SSP, Huntley MH, Durand NC, Stamenova EK, Bochkov ID, Robinson JT, et al. A 3d map of the human genome at kilobase resolution reveals principles of chromatin looping. Cell. 2014;159:1665–80. 25497547 10.1016/j.cell.2014.11.021 PMC5635824

[15] Dixon JR, Selvaraj S, Yue F, Kim A, Li Y, Shen Y, Hu M, Liu JS, Ren B. Topological domains in mammalian genomes identified by analysis of chromatin interactions. Nature. 2012;485:376–80. 22495300 10.1038/nature11082 PMC3356448

[16] Dekker J, Misteli T. (2015) Long-range chromatin interactions. Cold Spring Harb Perspect Biol, 7. 10.1101/cshperspect.a019356 PMC4588061 26430217

[17] Nakato R, Sakata T. Methods for ChIP-seq analysis: a practical workflow and advanced applications. Methods. 2021;187:44–53. 32240773 10.1016/j.ymeth.2020.03.005

[18] Ma S, Zhang Y. (2020) Profiling chromatin regulatory landscape: insights into the development of ChIP-seq and ATAC-seq. Mol Biomed, 1. 10.1186/s43556-020-00009-w PMC7546943 34765994

[19] Burgers TCQ, Vlijm R. Fluorescence-based super-resolution-microscopy strategies for chromatin studies. Chromosoma. 2023;132:191–209. 37000292 10.1007/s00412-023-00792-9 PMC10356683

[20] Cremer T, Cremer M. Chromosome territories. Cold Spring Harb Perspect Biol. 2010. 10.1101/cshperspect.a003889. 20300217 10.1101/cshperspect.a003889 PMC2829961

[21] Xu J, Liu Y. (2021) Probing chromatin compaction and its epigenetic States in situ with Single-Molecule Localization-Based Super-Resolution microscopy. Front Cell Dev Biol, 9. 10.3389/fcell.2021.653077 PMC8222792 34178982

[22] Xu J, Ma H, Jin J, Uttam S, Fu R, Huang Y, et al. Super-resolution imaging of higher-order chromatin structures at different epigenomic states in single mammalian cells. Cell Rep. 2018;24:873–82. 30044984 10.1016/j.celrep.2018.06.085 PMC6154382

[23] Cremer M, Schmid VJ, Kraus F, Markaki Y, Hellmann I, Maiser A, Leonhardt H, Stamatoyannopoulos JS,J. and, Cremer T. (2017) Initial high-resolution microscopic mapping of active and inactive regulatory sequences proves non-random 3D arrangements in chromatin domain clusters. Epigenetics Chromatin, 10. 10.1186/s13072-017-0146-0 PMC5547466 28784182

[24] Xu J, Zhou D, Deng D, Li J, Chen C, Liao X, Chen G, Heng PA. (2022) Deep Learning in Cell Image Analysis. *Intelligent Computing*, 2022.

[25] Heckenbach I, Mkrtchyan GV, Ezra MB, Bakula D, Madsen JS, Nielsen MH, et al. Nuclear morphology is a deep learning biomarker of cellular senescence. Nature Aging. 2022;2:742–55. 37118134 10.1038/s43587-022-00263-3 PMC10154217

[26] Azuma T, Kei T. Super-resolution spinning-disk confocal microscopy using optical photon reassignment. Opt Express. 2015;23:15003. 26072856 10.1364/OE.23.015003

[27] LaFoya B, Prehoda KE. (2021) Actin-dependent membrane polarization reveals the mechanical nature of the neuroblast Polarity cycle. Cell Rep, 35. 10.1016/j.celrep.2021.109146 PMC8174105 34010656

[28] Verth F, Fairn GD. Super-resolution spinning-disk confocal microscopy using optical photon reassignment (SoRa) to visualize the actin cytoskeleton in macrophages. Methods Mol Biol. 2023;2692:79–90. 37365462 10.1007/978-1-0716-3338-0_6

[29] Ecard J, Lian YL, Divoux S, Gouveia Z, Vigne E, Perez F, Boncompain G. Lysosomal membrane proteins LAMP1 and LIMP2 are segregated in the golgi apparatus independently of their clathrin adaptor binding motif. Mol Biol Cell. 2024;35:42–3. 10.1091/mbc.E23-06-0251 PMC10916873 38231876

[30] Jani RA, Cicco AD, Keren-Kaplan T, Vale-Costa S, Hamaoui D, Hurbain I, et al. PI4P and BLOC-1 remodel endosomal membranes into tubules. J Cell Biol. 2022;221:e202110132. 36169638 10.1083/jcb.202110132 PMC9524204

[31] Zhanghao K, Li M, Chen X, Liu W, Li T, Wang Y, et al. Fast segmentation and multiplexing imaging of organelles in live cells. Nat Commun. 2025;16:2769. 40118840 10.1038/s41467-025-57877-5 PMC11928634

[32] Sarnataro R, Velasco CD, Monaco N, Kempf A, Miesenböck G. Mitochondrial origins of the pressure to sleep. Nature. 2025;645:722–8. 40670797 10.1038/s41586-025-09261-y PMC12443607

[33] Singh D, Gupta S, Singh I, Morsy MA, Nair AB, Ahmed, A.S.F. (2021) Hidden Pharmacological activities of valproic acid: A new insight. Biomed Pharmacotherapy, 142. 10.1016/j.biopha.2021.112021 34463268

[34] Ibrahim TS, Sheha TA, Abo-Dya NE, AlAwadh MA, Alhakamy NA, Abdel-Samii ZK, Panda SS, Abuo-Rahma GEDA, Mohamed MFA. (2020) Design, synthesis and anticancer activity of novel valproic acid conjugates with improved histone deacetylase (HDAC) inhibitory activity. Bioorg Chem, 99. 10.1016/j.bioorg.2020.103797 32247939

[35] Marchion DC, Bicaku E, Daud AI, Sullivan DM, Munster PN. Valproic acid alters chromatin structure by regulation of chromatin modulation proteins. Cancer Res. 2005;65:3815–22. 15867379 10.1158/0008-5472.CAN-04-2478

[36] Duenas-Gonzalez A, Candelaria M, Perez-Plascencia C, Perez-Cardenas E, de la Cruz-Hernandez E, Herrera LA. Valproic acid as epigenetic cancer drug: preclinical, clinical and transcriptional effects on solid tumors. Cancer Treat Rev. 2008;34:206–22. 18226465 10.1016/j.ctrv.2007.11.003

[37] Aroosa M, Malik JA, Ahmed S, Bender O, Ahemad N, Anwar S. The evidence for repurposing anti-epileptic drugs to target cancer. Mol Biol Rep. 2023;50:7667–80. 37418080 10.1007/s11033-023-08568-1 PMC10460753

[38] Marano D, Fioriniello S, D’esposito M, Della Ragione F. Transcriptomic and epigenomic landscape in rett syndrome. Biomolecules. 2021. 10.3390/biom11070967. 34209228 10.3390/biom11070967 PMC8301932

[39] Okita K, Yamakawa T, Matsumura Y, Sato Y, Amano N, Watanabe A, Goshima N, Yamanaka S. An efficient nonviral method to generate integration-free human-induced pluripotent stem cells from cord blood and peripheral blood cells. Stem Cells. 2013;31:458–66. 23193063 10.1002/stem.1293

[40] Nakagawa M, Taniguchi Y, Senda S, et al. A novelefficient feeder-free culture system for the derivation of human induced pluripotent stem cells. Sci Rep. 2014;4:3594. . 10.1038/srep03594 24399248 10.1038/srep03594 PMC3884228

[41] He K, Gkioxari G, Dollár P, Girshick R. Mask R-CNN. IEEE Trans Pattern Anal Mach Intell. 2017;42:386–97. 10.1109/TPAMI.2018.2844175 29994331

[42] Johnson JW. (2018) Adapting Mask-RCNN for automatic nucleus segmentation. 10.1007/978-3-030-17798-0

[43] Caicedo JC, Goodman A, Karhohs KW, Cimini BA, Ackerman J, Haghighi M, et al. Nucleus segmentation across imaging experiments: the 2018 Data Science Bowl. Nat Methods. 2019;16:1247–53. 31636459 10.1038/s41592-019-0612-7 PMC6919559

[44] Bradski G. The Opencv library. Dr Dobb’s Journal: Softw Tools Prof Program. 2000;25:120–3.

[45] Paszke A, Gross S, Massa F, Lerer A, Bradbury Google J, Chanan G, Killeen T, Lin Z, Gimelshein N, Antiga L et al. (2019) PyTorch: an imperative Style, High-Performance deep learning library. Adv Neural Inf Process Syst, 32.

[46] Lin T-Y, Maire M, Belongie S, Bourdev L, Girshick R, Hays J, Perona P, Ramanan D, Zitnick CL, Dollár P. (2015) Microsoft COCO: Common Objects in Context. *Proceedings of the IEEE Computer Society Conference on Computer Vision and Pattern Recognition*.

[47] Wang H, Wang Z, Du M, Yang F, Zhang Z, Ding S, Mardziel P, Hu X. Score-CAM: Score-Weighted visual explanations for convolutional neural networks. IEEE Comput Soc Conf Comput Vis Pattern Recognit Workshops. 2019;2020–June:111–9.

[48] Gildenblat J. and contributors (2021) PyTorch library for CAM methods.

[49] Pourakpour F, Szölgyén Á, Nateghi R, Gutman DA, Manthey D, Cooper LA. HistomicsTK: a Python toolkit for pathology image analysis algorithms. SoftwareX. 2025;31:102318. 41048402 10.1016/j.softx.2025.102318 PMC12494233

[50] Virtanen P, Gommers R, Oliphant TE, Haberland M, Reddy T, Cournapeau D, Burovski E, Peterson P, Bright WW,J., et al. SciPy 1.0: fundamental algorithms for scientific computing in python. Nat Methods. 2020;17:261–72. 32015543 10.1038/s41592-019-0686-2 PMC7056644

[51] Durand NC, Shamim MS, Machol I, Rao SSP, Huntley MH, Lander ES, et al. Juicer provides a one-click system for analyzing loop-resolution Hi-C experiments. Cell Syst. 2016;3:95–8. 27467249 10.1016/j.cels.2016.07.002 PMC5846465

[52] Heinz S, Benner C, Spann N, Bertolino E, Lin YC, Laslo P, et al. Simple combinations of lineage-determining transcription factors prime cis-regulatory elements required for macrophage and B cell identities. Mol Cell. 2010;38:576–89. 20513432 10.1016/j.molcel.2010.05.004 PMC2898526

[53] Wolff J, Rabbani L, Gilsbach R, Richard G, Manke T, Backofen R, et al. Galaxy hicexplorer 3: a web server for reproducible Hi-C, capture Hi-C and single-cell Hi-C data analysis, quality control and visualization. Nucleic Acids Res. 2020;48:W177-84. 32301980 10.1093/nar/gkaa220 PMC7319437

[54] Cresswell KG, Dozmorov MG. (2020) TADCompare: an R package for differential and Temporal analysis of topologically associated domains. Front Genet, 11. 10.3389/fgene.2020.00158 PMC7076128 32211023

[55] Chen Y, Chen Y, Shi C, Huang Z, Zhang Y, Li S, Li Y, Ye J, Yu C, Li Z, et al. SOAPnuke: A mapreduce acceleration-supported software for integrated quality control and preprocessing of high-throughput sequencing data. Gigascience. 2018;7:1–6. 29220494 10.1093/gigascience/gix120 PMC5788068

[56] Dobin A, Davis CA, Schlesinger F, Drenkow J, Zaleski C, Jha S, Batut P, Chaisson M, Gingeras TR. STAR: ultrafast universal RNA-seq aligner. Bioinformatics. 2013;29:15–21. 23104886 10.1093/bioinformatics/bts635 PMC3530905

[57] Putri GH, Anders S, Pyl PT, Pimanda JE, Zanini F. Analysing high-throughput sequencing data in python with HTSeq 2.0. Bioinformatics. 2022;38:2943–5. 35561197 10.1093/bioinformatics/btac166 PMC9113351

[58] Love MI, Huber W, Anders S. (2014) Moderated Estimation of fold change and dispersion for RNA-seq data with DESeq2. Genome Biol, 15. 10.1186/s13059-014-0550-8 PMC4302049 25516281

[59] Wu T, Hu E, Xu S, Chen M, Guo P, Dai Z, et al. clusterProfiler 4.0: a universal enrichment tool for interpreting omics data. Innovation (Camb). 2021. 10.1016/j.xinn.2021.100141. 34557778 10.1016/j.xinn.2021.100141 PMC8454663

[60] Langmead B, Salzberg SL. Fast gapped-read alignment with bowtie 2. Nat Methods. 2012;9:357–9. 22388286 10.1038/nmeth.1923 PMC3322381

[61] Ramírez F, Ryan DP, Grüning B, Bhardwaj V, Kilpert F, Richter AS, Heyne S, Dündar F, Manke T. deepTools2: a next generation web server for deep-sequencing data analysis. Nucleic Acids Res. 2016;44:W160–5. 27079975 10.1093/nar/gkw257 PMC4987876

[62] Danecek P, Bonfield JK, Liddle J, Marshall J, Ohan V, Pollard MO, Whitwham A, Keane T, McCarthy SA, Davies RM. Twelve years of samtools and BCFtools. Gigascience. 2021;10:1–4. 10.1093/gigascience/giab008 PMC7931819 33590861

[63] Feng J, Liu T, Qin B, Zhang Y, Liu XS. Identifying ChIP-seq enrichment using MACS. Nat Protoc. 2012;7:1728–40. 22936215 10.1038/nprot.2012.101 PMC3868217

[64] Stark R, Brown G, DiffBind. Differential binding analysis of ChIP-Seq peak data.

[65] Ernst J, Kellis M. Chromatin-state discovery and genome annotation with chromhmm. Nat Protoc. 2017;12:2478–92. 29120462 10.1038/nprot.2017.124 PMC5945550

[66] Zhu X, Qi C, Wang R, Lee JH, Shao J, Bei L, Xiong F, Nguyen PT, Li G, Krakowiak J et al. (2022) Acute depletion of human core nucleoporin reveals direct roles in transcription control but dispensability for 3D genome organization. Cell Rep, 41. 10.1016/j.celrep.2022.111576 PMC9744245 36323253

[67] Altemose N, Maslan A, Rios-Martinez C, Lai A, White JA, Streets A. µDamID: a microfluidic approach for joint imaging and sequencing of protein-DNA interactions in single cells. Cell Syst. 2020;11:354-366.e9. 33099405 10.1016/j.cels.2020.08.015 PMC7588622

[68] Gel B, Díez-Villanueva A, Serra E, Buschbeck M, Peinado MA, Malinverni R. RegioneR: an R/Bioconductor package for the association analysis of genomic regions based on permutation tests. Bioinformatics. 2016;32:289–91. 26424858 10.1093/bioinformatics/btv562 PMC4708104

[69] Thi P, Anh M. (2023) Overview of Class Activation Maps for Visualization Explainability.

[70] Rullens PMJ, Kind J. Attach and stretch: emerging roles for genome–lamina contacts in shaping the 3D genome. Curr Opin Cell Biol. 2021;70:51–7. 33360765 10.1016/j.ceb.2020.11.006

[71] Briand N, Collas P. (2020) Lamina-associated domains: peripheral matters and internal affairs. Genome Biol, 21. 10.1186/s13059-020-02003-5 PMC7114793 32241294

[72] Meng Q, Zhang W, Wang X, Jiao C, Xu S, Liu C, et al. Human forebrain organoids reveal connections between valproic acid exposure and autism risk. Transl Psychiatry. 2022. 10.1038/s41398-022-01898-x. 35351869 10.1038/s41398-022-01898-x PMC8964691

[73] Balasubramanian D, Pearson JF, Kennedy MA. Gene expression effects of lithium and valproic acid in a serotonergic cell line. Physiol Genomics. 2019;51:43–50. 30576260 10.1152/physiolgenomics.00069.2018

[74] Logan RW, Ozburn AR, Arey RN, Ketchesin KD, Winquist A, Crain A, et al. Valproate reverses mania-like behaviors in mice via preferential targeting of HDAC2. Mol Psychiatry. 2021;26:4066–84. 33235333 10.1038/s41380-020-00958-2 PMC8141541

[75] Feleke R, Jazayeri D, Abouzeid M, Powell KL, Srivastava PK, O’Brien TJ, Jones NC, Johnson MR. Integrative genomics reveals pathogenic mediator of valproate-induced neurodevelopmental disability. Brain. 2022;145:3832–42. 36071595 10.1093/brain/awac296 PMC9679160

[76] Liu Y, Di Y, Zheng Q, Qian Z, Fan J, Ren W, Wei Z, Tian Y. (2022) Altered expression of glycan patterns and glycan-related genes in the medial prefrontal cortex of the valproic acid rat model of autism. Front Cell Neurosci, 16. 10.3389/fncel.2022.1057857 PMC9772556 36568890

[77] Zhang R, Zhou J, Ren J, Sun S, Di Y, Wang H, et al. Transcriptional and splicing dysregulation in the prefrontal cortex in valproic acid rat model of autism. Reprod Toxicol. 2018;77:53–61. 29427782 10.1016/j.reprotox.2018.01.008

[78] Nazer E. To be or not be (in the LAD): emerging roles of lamin proteins in transcriptional regulation. Biochem Soc Trans. 2022;50:1035–44. 35437578 10.1042/BST20210858 PMC9162450

[79] Shevelyov YY, Nurminsky DI. The nuclear lamina as a gene-silencing hub. Curr Issues Mol Biol. 2012;14:27–38. 21795760

[80] Shah PP, Santini GT, Shen KM, Jain R. Interlincing chromatin organization and mechanobiology in laminopathies. Curr Cardiol Rep. 2023;25:307–14. 37052760 10.1007/s11886-023-01853-2 PMC10185580

[81] Attali R, Warwar N, Israel A, Gurt I, McNally E, Puckelwartz M, Glick B, Nevo Y, Ben-Neriah Z, Melki J. Mutation of SYNE-1, encoding an essential component of the nuclear lamina, is responsible for autosomal recessive arthrogryposis. Hum Mol Genet. 2009;18:3462–9. 19542096 10.1093/hmg/ddp290

[82] Ghosh S, Cuevas VC, Seelbinder B, Neu CP. Image-based elastography of heterochromatin and euchromatin domains in the deforming cell nucleus. Small. 2021. 10.1002/smll.202006109. 33448065 10.1002/smll.202006109 PMC7869959

[83] Shah R, Smith P, Purdie C, Quinlan P, Baker L, Aman P, et al. The prolyl 3-hydroxylases P3H2 and P3H3 are novel targets for epigenetic silencing in breast cancer. Br J Cancer. 2009;100:1687–96. 19436308 10.1038/sj.bjc.6605042 PMC2696763

[84] Li J. (2021) Dysregulated expression of claudins in cancer (Review). Oncol Lett, 22. 10.3892/ol.2021.12902 PMC8298996 34386063

[85] Lopardo T, Lo Iacono N, Marinari B, Giustizieri ML, Cyr DG, Merlo G, et al. Claudin-1 is a p63 target gene with a crucial role in epithelial development. PLoS One. 2008. 10.1371/journal.pone.0002715. 18648642 10.1371/journal.pone.0002715 PMC2453228

[86] Abascal F, Acosta R, Addleman NJ, Adrian J, Afzal V, Aken B, Akiyama JA, Jammal O, Al AH, Anderson SM, et al. Expanded encyclopaedias of DNA elements in the human and mouse genomes. Nat 2020. 2020;583:7818:583, 699–710. 10.1038/s41586-020-2493-4 PMC7410828 32728249

[87] Schmidt A, Zhang H, Cristina Cardoso M. MeCP2 and chromatin compartmentalization. Cells. 2020. 10.3390/cells9040878. 32260176 10.3390/cells9040878 PMC7226738

[88] Jiang Y, Fu X, Zhang Y, Wang SF, Zhu H, Wang WK, Zhang L, Wu P, Wong CCL, Li J, et al. Rett syndrome linked to defects in forming the MeCP2/Rbfox/LASR complex in mouse models. Nat Commun 2021. 2021;12:1:12, 1–16. 10.1038/s41467-021-26084-3 PMC8486766 34599184

[89] Kang MK, Mehrazarin S, Park NH, Wang CY. Epigenetic gene regulation by histone demethylases: emerging role in oncogenesis and inflammation. Oral Dis. 2017;23:709–20. 27514027 10.1111/odi.12569 PMC5493521

[90] Lee W, Yun JM, Woods R, Dunaway K, Yasui DH, Lasalle JM, Gong Q. MeCP2 regulates activity-dependent transcriptional responses in olfactory sensory neurons. Hum Mol Genet. 2014;23:6366–74. 25008110 10.1093/hmg/ddu358 PMC4222369

[91] Astolfi A, Fiore M, Melchionda F, Indio V, Bertuccio SN, Pession A. BCOR involvement in cancer. Epigenomics. 2019;11:835–55. 31150281 10.2217/epi-2018-0195 PMC6595546

[92] Wang GS, Hong CJ, Yen TY, Huang HY, Ou Y, Huang TN, et al. Transcriptional modification by a CASK-interacting nucleosome assembly protein. Neuron. 2004;42:113–28. 15066269 10.1016/s0896-6273(04)00139-4

[93] Pascual-Alonso A, Xiol C, Smirnov D, Kopajtich R, Prokisch H, Armstrong J. (2023) Identification of molecular signatures and pathways involved in Rett syndrome using a multi-omics approach. Hum Genomics, 17. 10.1186/s40246-023-00532-1 PMC10503149 37710353

[94] Rahman S, Roussos P. The 3D genome in brain development: an exploration of molecular mechanisms and experimental methods. Neurosci Insights. 2024. 10.1177/26331055241293455. 39494115 10.1177/26331055241293455 PMC11528596

[95] Dekker J, Mirny L. The 3d genome as moderator of chromosomal communication. Cell. 2016;164:1110–21. 26967279 10.1016/j.cell.2016.02.007 PMC4788811

[96] Super-resolution microscopy at its sharpest. (2025) *Nat Photonics*, 19, 219.

[97] Farhy C, Hariharan S, Ylanko J, Orozco L, Zeng FY, Pass I, Ugarte F, Forsberg C, Huang CT, Andrews D et al. (2019) Improving drug discovery using image-based multiparametric analysis of the epigenetic landscape. Elife, 8. 10.7554/eLife.49683 PMC6908434 31637999

[98] Chandrasekaran SN, Ceulemans H, Boyd JD, Carpenter AE. Image-based profiling for drug discovery: due for a machine-learning upgrade? Nat Rev Drug Discov. 2021;20:145–59. 33353986 10.1038/s41573-020-00117-w PMC7754181

[99] Moses L, Pachter L. Museum of Spatial transcriptomics. Nat Methods 2022. 2022;19:5:19, 534–46. 10.1038/s41592-022-01409-2 35273392

[100] Zhuang X. Spatially resolved single-cell genomics and transcriptomics by imaging. Nat Methods. 2021;18:18. 33408406 10.1038/s41592-020-01037-8 PMC9805800

[101] Jiang X, Wang S, Guo L, Zhu B, Wen Z, Jia L, Xu L, Xiao G, Li Q. iIMPACT: integrating image and molecular profiles for Spatial transcriptomics analysis. Genome Biology 2024. 2024;25:1:25, 147. 10.1186/s13059-024-03289-5 PMC11514947 38844966

[102] Su JH, Zheng P, Kinrot SS, Bintu B, Zhuang X. Genome-scale imaging of the 3d organization and transcriptional activity of chromatin. Cell. 2020;182:1641. 32822575 10.1016/j.cell.2020.07.032 PMC7851072

[103] Takei Y, Yun J, Zheng S, Ollikainen N, Pierson N, White J, et al. Integrated spatial genomics reveals global architecture of single nuclei. Nature. 2021;590:344. 33505024 10.1038/s41586-020-03126-2 PMC7878433

[104] Takei Y, Yang Y, White J, Goronzy IN, Yun J, Prasad M, Ombelets LJ, Schindler S, Bhat P, Guttman M, et al. Spatial multi-omics reveals cell-type-specific nuclear compartments. Nat 2025. 2025;641:8064:641, 1037–47. 10.1038/s41586-025-08838-x 40205045

[105] Payne AC, Chiang ZD, Reginato PL, Mangiameli SM, Murray EM, Yao CC, Markoulaki S, Earl AS, Labade AS, Jaenisch R, et al. In situ genome sequencing resolves DNA sequence and structure in intact biological samples. Science. 2020;371:eaay3446. 33384301 10.1126/science.aay3446 PMC7962746

[106] Labade AS, Chiang ZD, Comenho C, Reginato PL, Payne AC, Earl AS, Shrestha R, Duarte FM, Habibi E, Zhang R et al. (2025) Expansion insitu genome sequencing links nuclear abnormalities to aberrant chromatin regulation. *Science (1979)*, 389. 10.1126/science.adt2781 40440430

[107] Felisbino MB, Alves da Costa T, Gatti MSV, Mello MLS. Differential response of human hepatocyte chromatin to HDAC inhibitors as a function of microenvironmental glucose level. J Cell Physiol. 2016;231:2257–65. 26888775 10.1002/jcp.25343

[108] Stephens AD, Banigan EJ, Marko JF. Separate roles for chromatin and lamins in nuclear mechanics. Nucleus. 2018;9:119–24. 29227210 10.1080/19491034.2017.1414118 PMC5973264

[109] Bártová E, Pacherník J, Harničarová A, Kovařík A, Kovaříková M, Hofmanová J, et al. Nuclear levels and patterns of histone H3 modification and HP1 proteins after inhibition of histone deacetylases. J Cell Sci. 2005;118:5035–46. 16254244 10.1242/jcs.02621

[110] Taddei A, Maison C, Roche D, Almouzni G. Reversible disruption of pericentric heterochromatin and centromere function by inhibiting deacetylases. Nat Cell Biol. 2001;3:114–20. 11175742 10.1038/35055010

[111] Gilchrist S, Gilbert N, Perry P, Bickmore WA. Nuclear organization of centromeric domains is not perturbed by inhibition of histone deacetylases. Chromosom Res. 2004;12:505–16. 10.1023/B:CHRO.0000034892.64739.ff 15254368

[112] Lilja T, Wallenborg K, Björkman K, Albåge M, Eriksson M, Lagercrantz H, Rohdin M, Hermanson O. Novel alterations in the epigenetic signature of MeCP2-targeted promoters in lymphocytes of Rett syndrome patients. Epigenetics. 2013;8:246–51. 23348913 10.4161/epi.23752 PMC3669117

[113] Boxer LD, Renthal W, Greben AW, Whitwam T, Silberfeld A, Stroud H, Li E, Yang MG, Kinde B, Griffith EC, et al. MeCP2 represses the rate of transcriptional initiation of highly methylated long genes. Mol Cell. 2019;77:294. 31784358 10.1016/j.molcel.2019.10.032 PMC6982532

[114] Clemens AW, Wu DY, Moore JR, Christian DL, Zhao G, Gabel HW. MeCP2 represses enhancers through chromosome topology-associated DNA methylation. Mol Cell. 2019;77:279. 31784360 10.1016/j.molcel.2019.10.033 PMC6980697

[115] Kernohan KD, Vernimmen D, Gloor GB, Bérubé NG. Analysis of neonatal brain lacking ATRX or MeCP2 reveals changes in nucleosome density, CTCF binding and chromatin looping. Nucleic Acids Res. 2014;42:8356–68. 24990380 10.1093/nar/gku564 PMC4117782

[116] Wang L, Hu M, Zuo MQ, Zhao J, Wu D, Huang L, et al. Rett syndrome-causing mutations compromise MeCP2-mediated liquid–liquid phase separation of chromatin. Cell Res. 2020;30(5):393–407. 32111972 10.1038/s41422-020-0288-7 PMC7196128

[117] Li CH, Coffey EL, Dall’Agnese A, Hannett NM, Tang X, Henninger JE, Platt JM, Oksuz O, Zamudio AV, Afeyan LK, et al. MeCP2 links heterochromatin condensates and neurodevelopmental disease. Nature. 2020;586:440–4. 32698189 10.1038/s41586-020-2574-4 PMC7735819

[118] Kaufmann WE, Jarrar MH, Wang JS, Lee YJM, Reddy S, Bibat G, et al. Histone modifications in Rett syndrome lymphocytes: a preliminary evaluation. Brain Dev. 2005;27:331–9. 16023547 10.1016/j.braindev.2004.09.005

[119] Urdinguio RG, Pino I, Ropero S, Fraga MF, Esteller M. Histone H3 and H4 modification profiles in a Rett syndrome mouse model. Epigenetics. 2007;2:11–4. 17965622 10.4161/epi.2.1.3698

