# Supplementary material for: Image-based epigenetic profiling with deep learning and high-speed super-resolution microscopy
Source: Epigenetics Chromatin. 2026 Feb 28;19:15. doi: 10.1186/s13072-026-00662-5 (PMC13059569; doi:10.1186/s13072-026-00662-5)
Supplement: Supplementary file 2 — Supplementary Material 2 [file 13072_2026_662_MOESM2_ESM.pdf]

Supplementary Material

Image-based epigenetic profiling with deep learning and high-speed super-resolution microscopy

Yicheng Wang[1†], Nur Syatila Ab Ghani[2†], Munmee Dutta[3], Shungo Adachi[4], Kaoru Katoh[5], Masakazu Namihira[6,7]*, Toutai Mitsuyama[3], Yutaka Saito[1,2,3]*

[1] Graduate School of Frontier Sciences, The University of Tokyo, 5-1-5 Kashiwanoha, Kashiwa, Chiba 277-0882, Japan

[2] Department of Data Science, School of Frontier Engineering, Kitasato University, 1-15-1 Kitazato, Minami-ku, Sagamihara, Kanagawa 252-0373, Japan

[3] Artificial Intelligence Research Center, National Institute of Advanced Industrial Science and Technology (AIST), 2-4-7 Aomi, Koto-ku, Tokyo 135-0064, Japan

[4] Department of Proteomics, National Cancer Center Research Institute, Tsukiji 5-1-1, Chuo-ku, Tokyo 104-0045, Japan

[5] Exploratory Research Center on Life and Living Systems, National Institutes of Natural Sciences, 5-1 Higashiyama Myodaijicho, Okazaki, Aichi 444-8787, Japan

[6] Molecular Biosystems Research Institute, National Institute of Advanced Industrial Science and Technology (AIST), 1-1-1 Higashi, Tsukuba, Ibaraki 305-8566, Japan

[7] Laboratory of Neural Regeneration and Brain Repair, Division of Biological Science, Graduate School of Science and Technology, Nara Institute of Science and Technology (NAIST), 8916-5 Takayama-cho, Ikoma, Nara, 630-0192, Japan

† These authors contributed equally to this work.

* To whom correspondence should be addressed. Email: saito.yutaka@kitasato-u.ac.jp.

Correspondence may also be addressed to Masakazu Namihira, Email: m-namihira@aist.go.jp.

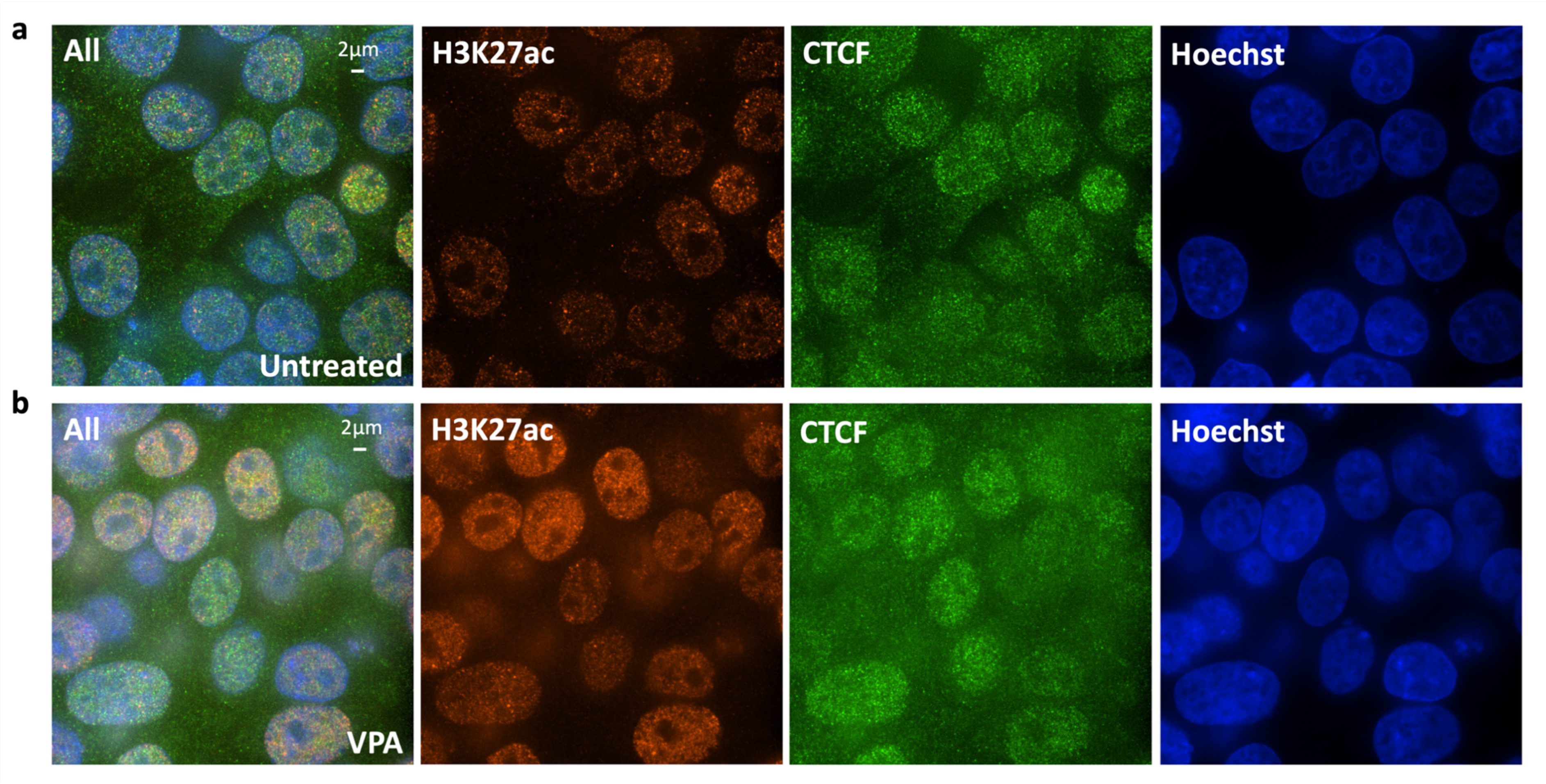

Figure S1. Example images of cells captured using the high-speed super-resolution microscopy SoRa. (a) Untreated cells under various staining conditions. **(b)** VPA-treated cells under various staining conditions.

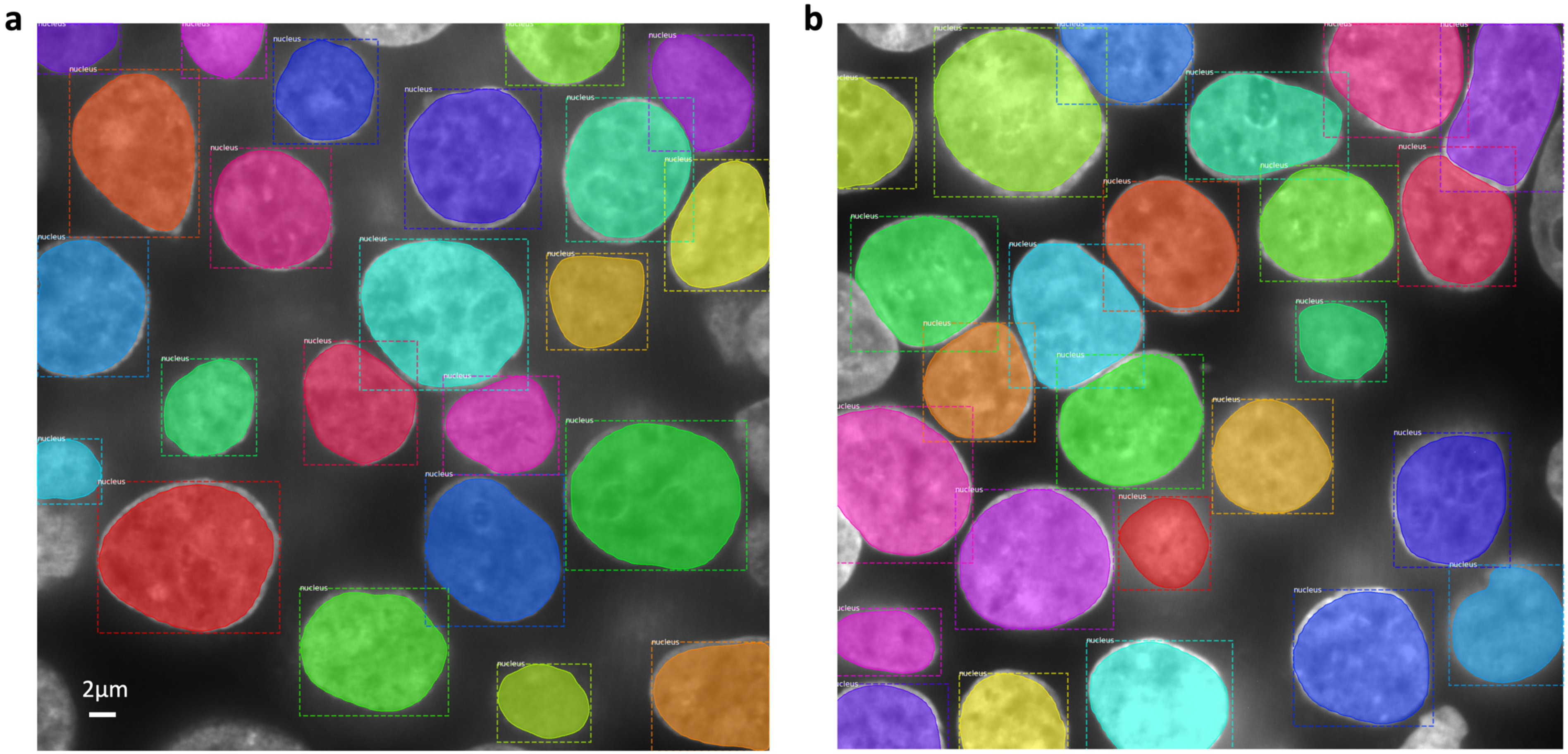

Figure S2. Examples of nucleus segmentation by Mask RCNN. The varied color schemes represent different nucleus that have been segmented. **(a)** Example segmentation result from an untreated cell image. (**b**) Example segmentation result from a VPA-treated cell image.

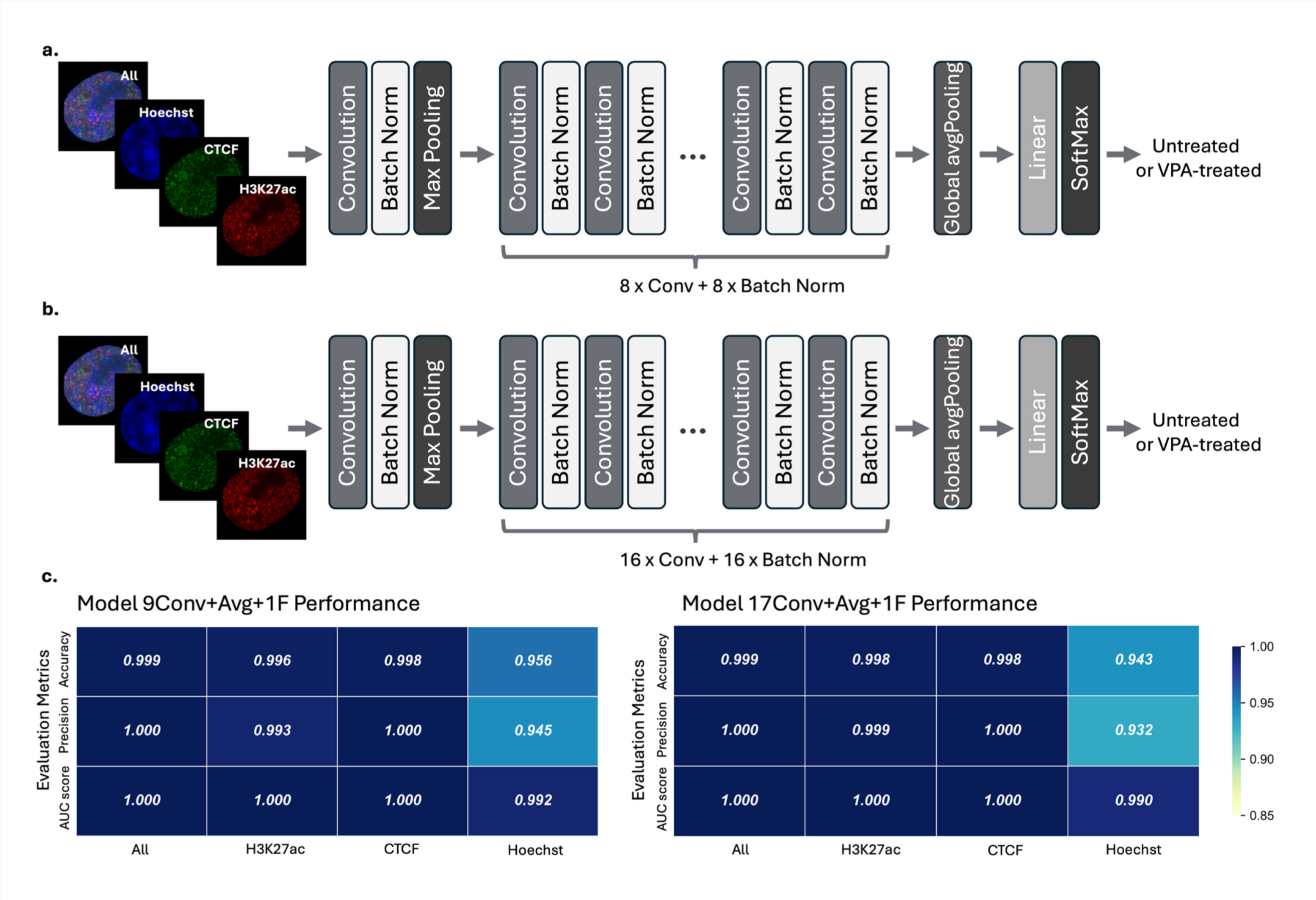

Figure S3. Two additional models employed in this study. (a) 9Conv+Avg+1F model, which includes an average pooling layer. **(b)** 17Conv+Avg+1F model, also featuring an average pooling layer. **(c)** Performance of these two models.

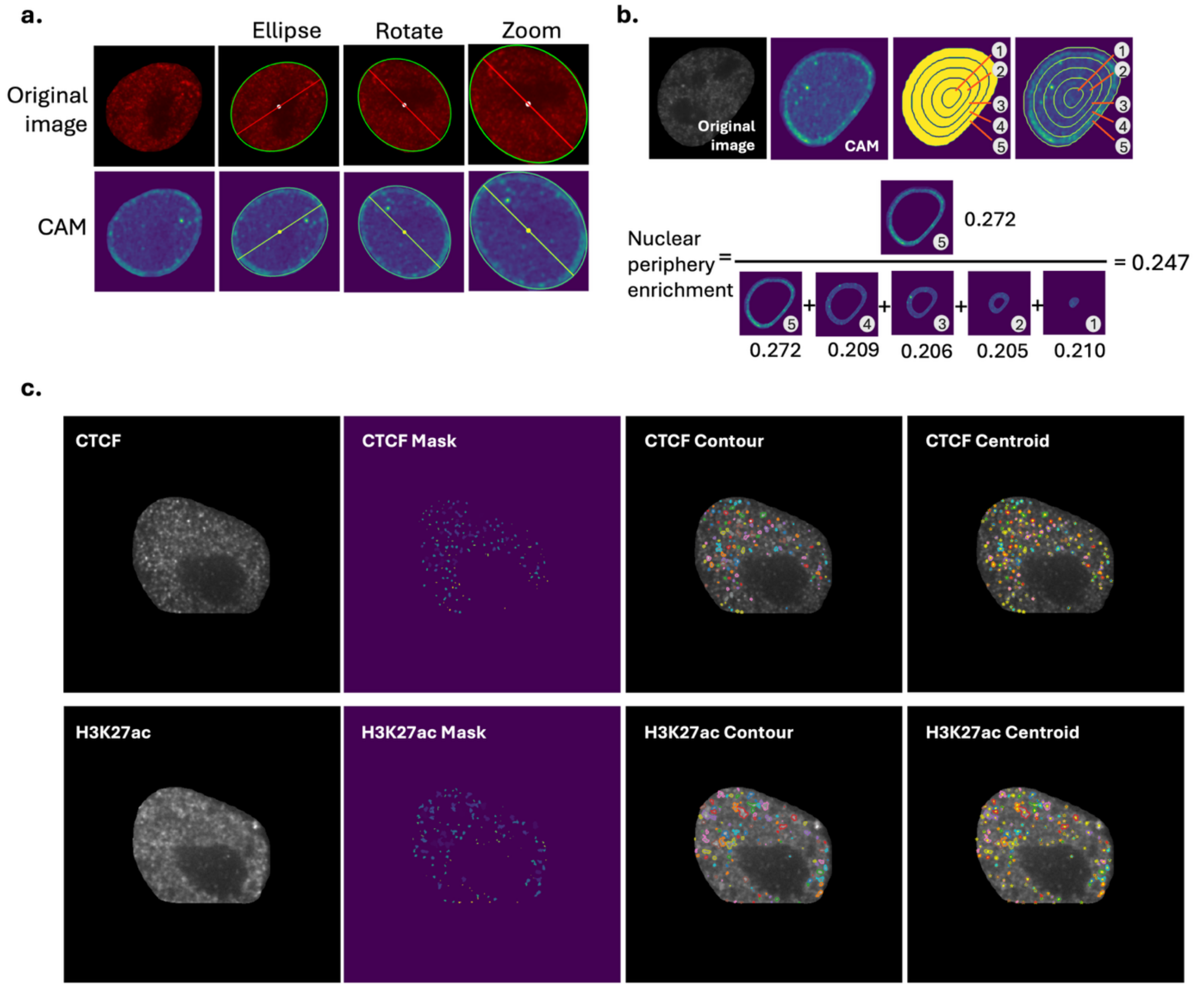

Figure S4. Overview of CAM heatmap processing and puncta segmentation methods. (a) Workflow for processing Score-CAM heatmaps, including alignment to fitted nuclear ellipses for generating average heatmaps. **(b)** Procedure for calculating nuclear periphery enrichment (Methods). **(c)** Examples of puncta segmentation results for CTCF (top) and H3K27ac (bottom), with corresponding puncta centroids indicated.

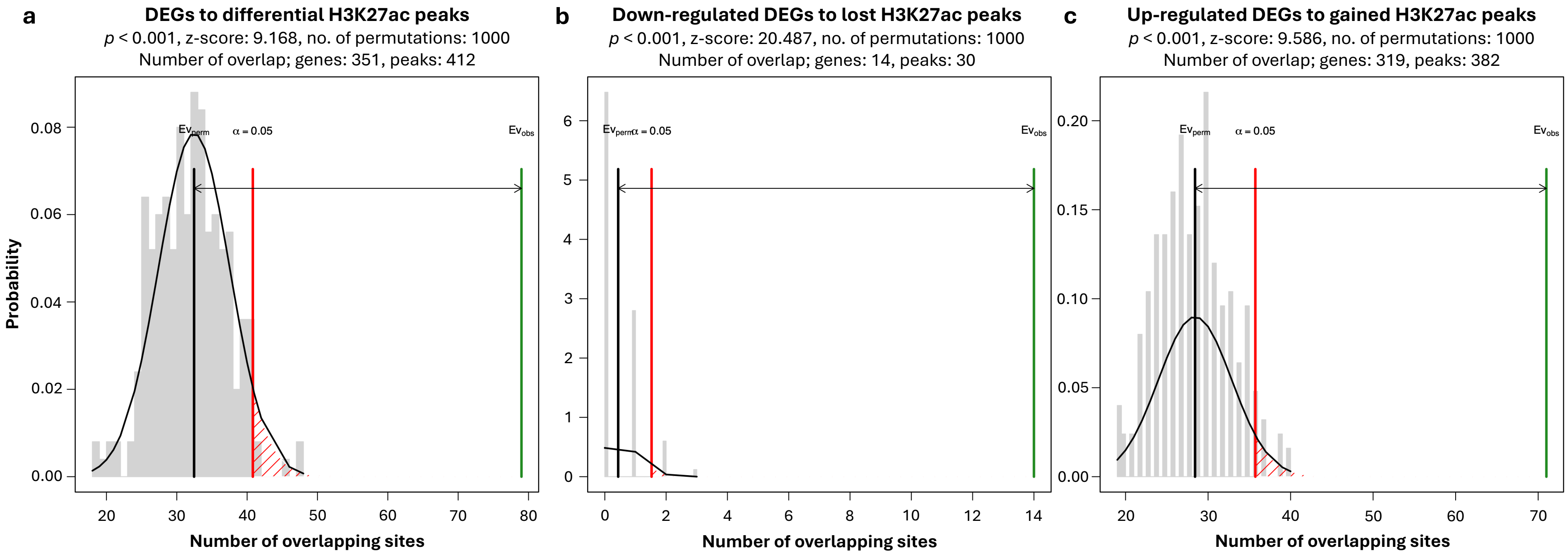

Figure S5. Overlap between differentially expressed genes (DEGs) and differential H3K27ac ChIP-seq peaks analyzed by permutation test using RegioneR. **(a)** DEGs and differential H3K27ac peaks. **(b)** Down-regulated DEGs enriched in lost peaks (peaks with significantly higher enrichment in untreated cells). **(c)** Up-regulated DEGs were enriched in gained peaks (peaks with significantly higher enrichment in VPA-treated cells). The x-axis represents the number of overlapping sites, and the y-axis represents the probability density, showing the estimated null distribution from 1000 permutations of randomly selected regions from the two region sets. The red vertical line indicates the significance threshold of $p = 0.05$. The green line denotes the observed number of overlapping regions ($p < 0.001$).

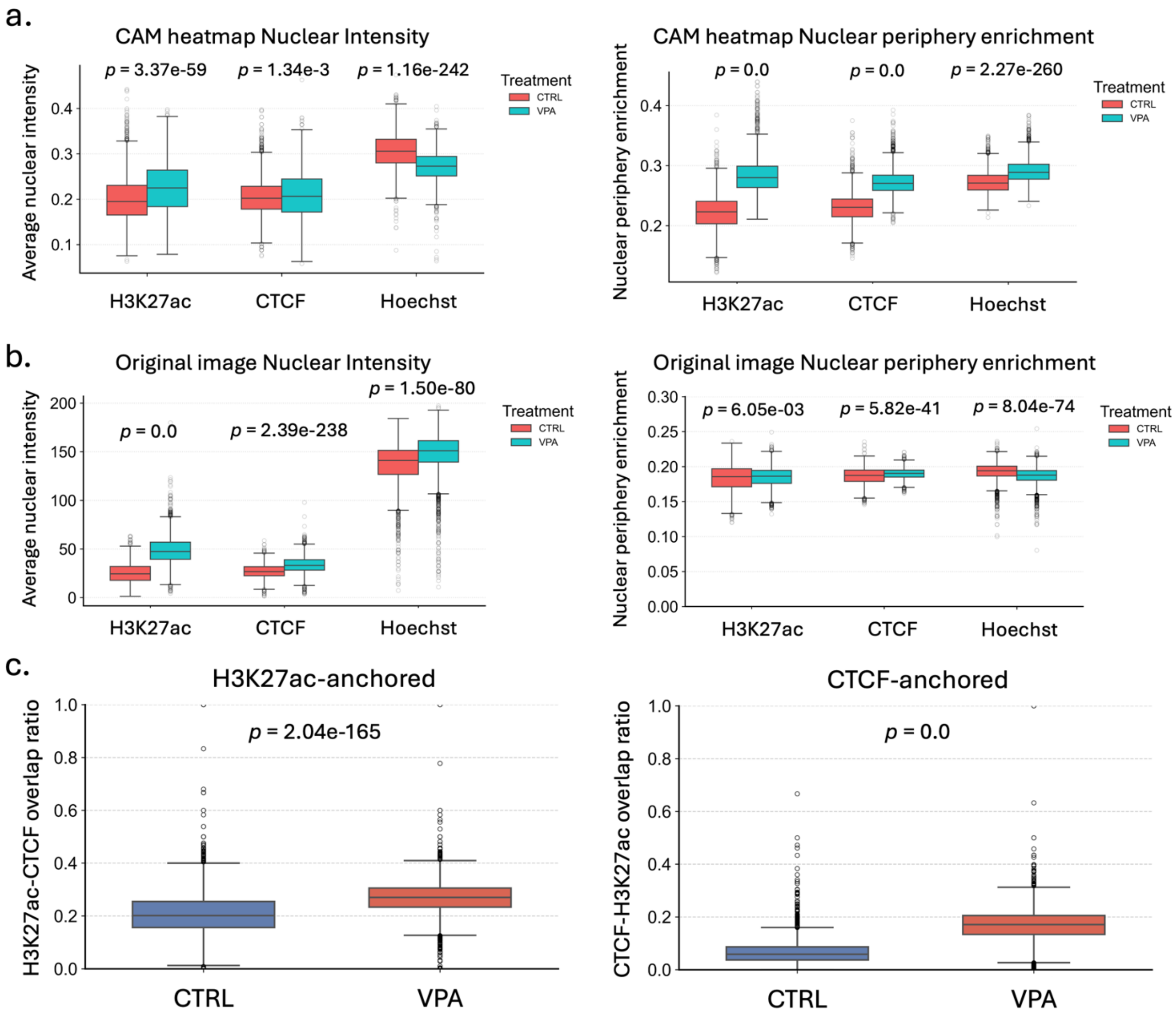

Figure S6. Image-based nuclear features and chromatin puncta overlap of VPA treatment in HEK293T cells. (a). Score-CAM heatmap metrics of nuclear intensity (left) and nuclear periphery enrichment (right) for H3K27ac-only, CTCF-only and Hoechst-only models. **(b)** Original image metrics of nuclear intensity (left) and nuclear periphery enrichment (right) for H3K27ac-only, CTCF-only and Hoechst-only models. **(c)** Puncta overlap ratio based on H3K27ac-anchored puncta (left) and CTCF-anchored puncta (right).

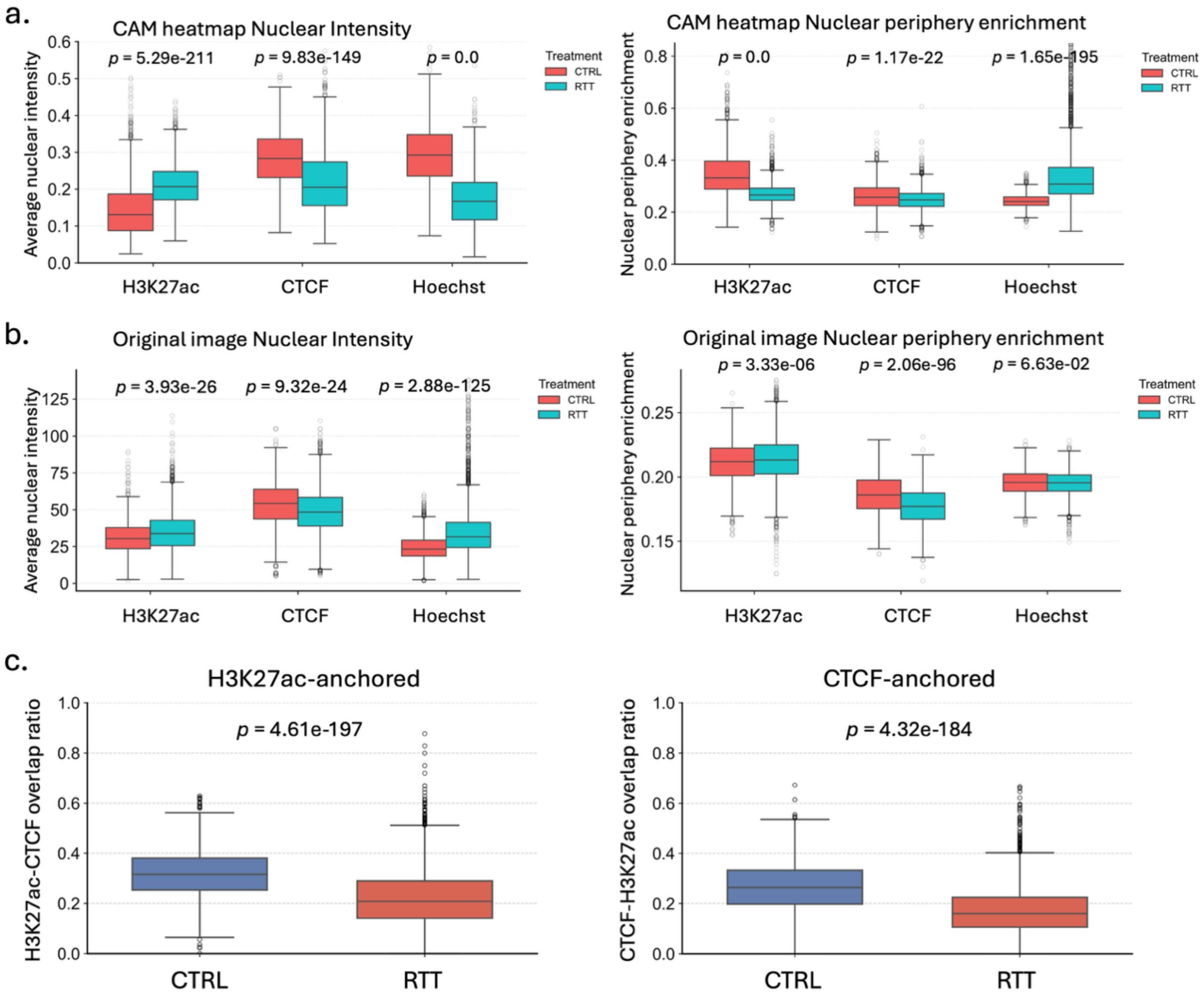

Figure S7. Image-based nuclear features and chromatin puncta overlap of RTT in iPS cells. (a). Score-CAM heatmap metrics of nuclear intensity (left) and nuclear periphery enrichment (right) for H3K27ac-only, CTCF-only and Hoechst-only models. **(b)** Original image metrics of nuclear intensity (left) and nuclear periphery enrichment (right) for H3K27ac-only, CTCF-only and Hoechst-only models. **(c)** Puncta overlap ratio based on H3K27ac-anchored puncta (left) and CTCF-anchored puncta (right).
